# Supplementary material for: Air quality improvement and cognitive decline in community-dwelling older women in the United States: A longitudinal cohort study
Source: PLoS Med. 2022 Feb 3;19(2):e1003893. doi: 10.1371/journal.pmed.1003893 (PMC8812844; doi:10.1371/journal.pmed.1003893)
Supplement: S1 Text — WHI, Women’s Health Initiative. (DOCX) [file pmed.1003893.s002.docx]

**S1 Text.** **Covariates Assessed at Women’s Health Initiative (WHI) Inception**

Participants completed structured questionnaires at WHI inception to provide information on demographics (geographic region, age, and race/ethnicity), socioeconomic factors (education, family income, and employment status), and lifestyle factors (smoking status, alcohol intake, and physical activity).

Geographic region was a 4-categorical variable (Northeast, South, Midwest, and West) based on U.S. Census definition, according to the residence at the time of WHI-Hormone Therapy clinical trial randomization.

Highest grade finished in school was collected and grouped into a three-categorical education variable (≤ High school or general educational development; > High school but < 4 years of college; or ≥ 4 years of college). Total family income was grouped into five categories (< $9,999; $10,000-$34,999; $35,000-$74,999; ≥ $75,000; or unknown). Based on current and past employment information, employment status was grouped into three categories: currently employed, not working, or retired.

Smoking status was coded as never, past, or current smoker. According to past and current use of alcohol and number of current servings (12 oz of beer, 6 oz of wine, and 1.5 oz of liquor) per week, alcohol intake was categorized as non-drinker, past-drinker, < 1 drink per day, or ≥ 1 drink per day. Physical activity was coded based on number of episodes per week of moderate (e.g., biking outdoors, using an exercise machine, calisthenics, easy swimming, popular or folk dancing) and strenuous (e.g., aerobics, aerobic dancing, jogging, tennis, swimming laps) recreational physical activity of ≥ 20 minutes duration, which was categorized as no activity, some activity, 2-4 episodes per week, or > 4 episodes per week.
