# Supplementary material for: Air quality improvement and cognitive decline in community-dwelling older women in the United States: A longitudinal cohort study
Source: PLoS Med. 2022 Feb 3;19(2):e1003893. doi: 10.1371/journal.pmed.1003893 (PMC8812844; doi:10.1371/journal.pmed.1003893)
Supplement: S2 Text — (DOCX) [file pmed.1003893.s003.docx]

**S2 Text.** **Assessment of U.S. Census Tract-Level Socioeconomic Characteristics of Residential Neighborhood**

Data from the U.S. Census of Population and Housing 2000 Summary File 3 [1] or their 5-year analogs from the American Community Survey 2005-2009 to 2013-2017 [2] were temporally matched to the geocoded participant addresses [3,4]. Each measure had been aggregated at the U.S. Census tract level (i.e., the lowest geographic level historically associated with accurate and reliable assignment of Federal Information Processing System codes) [4]. Neighborhood socioeconomic status was characterized using U.S. census track-level measures in six domains: 1) natural log-transformed median household income (in dollars); 2) natural log transformed median value (in dollars) of owner-occupied housing units; 3) percentage of households receiving interest, dividends or net rental income; 4) percentage of adults aged 25+ with a high school degree; 5) percentage of adults aged 25+ with a college degree; and 6) percentage of civilian population aged 16+ with professional, managerial, or executive occupations. The six variables were first standardized using the corresponding population-specific mean and standard deviation and then summed up, yielding a neighborhood socioeconomic status summary z score [5].

Supplemental References

1. U.S. Census Bureau, Inter-university Consortium for Political and Social Research. Census of Population and Housing, 2000 [United States]: Selected Subsets From Summary File 3. Inter-university Consortium for Political and Social Research [distributor]; 2006.

2. U.S. Census Bureau. American Community Survey 5-Year Estimates. Social Explorer.

3. Whitsel EA, Rose KM, Wood JL, Henley AC, Liao D, Heiss G. Accuracy and repeatability of commercial geocoding. Am J Epidemiol. 2004;160(10):1023-9. doi: 10.1093/aje/kwh310. PubMed PMID: 15522859.

4. Whitsel EA, Quibrera PM, Smith RL, Catellier DJ, Liao D, Henley AC, et al. Accuracy of commercial geocoding: assessment and implications. Epidemiol Perspect Innov. 2006;3:8. Epub 2006/07/20. doi: 10.1186/1742-5573-3-8. PubMed PMID: 16857050; PubMed Central PMCID: PMCPMC1557664.

5. Diez Roux AV, Merkin SS, Arnett D, Chambless L, Massing M, Nieto FJ, et al. Neighborhood of residence and incidence of coronary heart disease. N Engl J Med. 2001;345(2):99-106. doi: 10.1056/NEJM200107123450205. PubMed PMID: 11450679.
