# Supplementary material for: Air quality improvement and cognitive decline in community-dwelling older women in the United States: A longitudinal cohort study
Source: PLoS Med. 2022 Feb 3;19(2):e1003893. doi: 10.1371/journal.pmed.1003893 (PMC8812844; doi:10.1371/journal.pmed.1003893)
Supplement: S3 Text — WHIMS-ECHO, Women’s Health Initiative Memory Study-Epidemiology of Cognitive Health Outcomes. (DOCX) [file pmed.1003893.s004.docx]

**S3 Text.** **Assessment of Covariates at the Women’s Health Initiative Memory Study-Epidemiology of Cognitive Health Outcomes (WHIMS-ECHO) Enrollment**

In addition to the self-reported history of cardiovascular disease (CVD) collected at the WHI inception, prospectively collected data on incident cardiovascular diseases (coronary heart disease; myocardial infarction; coronary revascularization; coronary angioplasty; coronary artery bypass graft; atrial fibrillation; stroke) were used to update the information on CVD histories prior to the WHIMS-ECHO enrollment. Hypertension at the WHIMS-ECHO enrollment was defined as having a history of hypertension at WHI inception or having an elevated blood pressure (systolic ≥ 140 or diastolic ≥ 90 mmHg) before the WHIMS-ECHO enrollment. Body mass index (BMI) was calculated using height and weight measured at the closest clinic visit before the WHIMS-ECHO enrollment. Lifestyle factors, including current smoking status (Yes vs. No), alcohol consumption (servings per week), and physical activity (number of episodes per week of moderate and strenuous recreational physical activity of ≥ 20 minutes), were collected using a questionnaire administered at the closest time before the WHIMS-ECHO enrollment.
