# Supplementary material for: Air quality improvement and cognitive decline in community-dwelling older women in the United States: A longitudinal cohort study
Source: PLoS Med. 2022 Feb 3;19(2):e1003893. doi: 10.1371/journal.pmed.1003893 (PMC8812844; doi:10.1371/journal.pmed.1003893)
Supplement: S4 Text — WHI, Women’s Health Initiative; WHIMS-ECHO, Women’s Health Initiative Memory Study-Epidemiology of Cognitive Health Outcomes. (DOCX) [file pmed.1003893.s005.docx]

**S4 Text.** **Assessment of longitudinal changes in covariates from the WHI inception to WHIMS-ECHO enrollment**

For continuous measures, including neighborhood socioeconomic characteristics, alcohol consumption, physical activity, and BMI, longitudinal changes were calculated by taking the difference from WHIMS-ECHO enrollment to WHI inception. For smoking status, change was categorized as no change, initiation of smoking, and cessation of smoking. Change in histories of hypertension or CVD were dichotomized as no vs. yes.
