# Supplementary material for: Air quality improvement and cognitive decline in community-dwelling older women in the United States: A longitudinal cohort study
Source: PLoS Med. 2022 Feb 3;19(2):e1003893. doi: 10.1371/journal.pmed.1003893 (PMC8812844; doi:10.1371/journal.pmed.1003893)
Supplement: S5 Text — APoE, Apolipoprotein E. (DOCX) [file pmed.1003893.s006.docx]

**S5 Text.** **APOE Genotype Data**

Genetic data were from a WHI core study (W63) resource derived from the genome-wide association study sample. APOE genotypes were assigned based on rs429358 and rs7412 genotype results from imputation and harmonization. The imputation was conducted using the 1000 Genomes Project reference panel and the MaCH algorithm, as implemented in Minimac (R^2^ = 0.98 for each SNP in the study population). Women with genotypes e2/2, e2/3 and e3/3 were combined as one group representing those without e4 allele, while women with genotypes e2/4, e3/4 and e4/4 were grouped together representing women with at least one copy of e4 allele in analysis.
