# Supplementary material for: Air quality improvement and cognitive decline in community-dwelling older women in the United States: A longitudinal cohort study
Source: PLoS Med. 2022 Feb 3;19(2):e1003893. doi: 10.1371/journal.pmed.1003893 (PMC8812844; doi:10.1371/journal.pmed.1003893)
Supplement: S6 Text — (DOCX) [file pmed.1003893.s007.docx]

**S6 Text.** **Equations of Three-Level Linear Mixed Effect Models**

We used a three-level linear mixed effect model to estimate the association between air quality improvement and cognitive function decline. Two outcome measures were considered: general cognitive status which was estimated by modified Telephone Interview for Cognitive Status (TICSm) or episodic memory that was assessed by California Verbal Learning Tests (CVLT). All models included a random intercept at both Women’s Health Initiative (WHI) clinic site and individual levels.

The equation to estimate the association between air quality improvement and cognitive function decline can be written as:

$$\boldsymbol{Equation A}:$$

$$Y_{ijk}=\alpha+\beta_{1}*\mathrm{AQ}_{improvement, ij}+\beta_{2}*\mathrm{Time}_{ijk}+\beta_{3}*\mathrm{Age}_{ij}+\beta_{4}*\mathrm{AQ}_{improvement, ij}*\mathrm{Time}_{ijk}$$

$$+\beta_{5}*\mathrm{Age}_{ij}*\mathrm{Time}_{ijk}+\sum_{p=1}^{P} \gamma_{p}*Z_{\mathrm{pij}}+a_{i}+a_{ij}+\epsilon_{ijk}$$

In this equation,$Y_{ijk}$ represents repeated measures of TICSm or CVLT, where *i* represents clinic site, *j* represents individual, and *k* represents the visit number since WHIMS-ECHO enrollment. The variable $\mathrm{AQ}_{improvement, ij}$ represents the air quality improvement measure for individual *j* at clinic site *i*. The $\mathrm{Time}_{ijk}$ variable represents year since WHIMS-ECHO enrollment for individual *j* in clinic site *i* at the *k^th^* visit. $\mathrm{Age}_{ij}$ represents age at WHIMS-ECHO enrollment. $Z_{1ij}, \ldots, Z_{Pij}$ represent all other individual-level covariates included in the model. $a_{i}\sim N(0,\delta^{2})$ is the random intercept at WHI clinic site level and $a_{ij}\sim N(0,\varphi^{2})$ is the random intercept at the individual level. $\epsilon_{ijk}\sim N(0,\sigma^{2})$ represents the error term. The coefficient $\beta_{4}$ is the association that we would like to estimate between air quality improvement and cognitive function decline after adjusting for all covariates. A product term of $\mathrm{Age}_{ij}$ and $\mathrm{Time}_{ijk}$ was also included in models to evaluate the association ($\beta_{5}$) between age and cognitive function decline. The ratio of $\beta_{4}$ over $\beta_{5}$ could be used to evaluate the aging-equivalent effect size for the association between air quality improvement and cognitive function decline.

The equation to estimate whether the association between air quality improvement and cognitive function decline differed by our effect modifiers can be written as:

$$\boldsymbol{Equation B}:$$

$$Y_{ijk}=\alpha^{'}+\beta_{1}^{'}*\mathrm{AQ}_{improvement, ij}+\beta_{2}^{'}*\mathrm{Time}_{ijk}+\beta_{3}^{'}*\mathrm{Age}_{ij}+\beta_{4}^{'}*\mathrm{AQ}_{improvement, ij}*\mathrm{Time}_{ijk}$$

$$+\beta_{5}^{'}*\mathrm{Age}_{ij}*\mathrm{Time}_{ijk}+\beta_{6}^{'}*\mathrm{EM}_{ij}+\beta_{7}^{'}*\mathrm{EM}_{ij}*\mathrm{AQ}_{improvement, ij}+\beta_{8}^{'}*\mathrm{EM}_{ij}*\mathrm{Time}_{ijk}$$

$$+\beta_{9}^{'}*\mathrm{EM}_{ij}*\mathrm{AQ}_{improvement, ij}*\mathrm{Time}_{ijk}+\sum_{p=1}^{P} \gamma_{p}^{'}*Z_{\mathrm{pij}}+a_{i}^{'}+a_{ij}^{'}+\epsilon_{ijk}^{'}$$

In this equation, a new variable $\mathrm{EM}_{ij}$ representing the effect modifier and its product term with $\mathrm{AQ}_{improvement, ij}$, $\mathrm{Time}_{ijk}$, or both were included. $a_{i}^{'}\sim N(0,{\delta^{'}}^{2})$ is the random intercept at the WHI clinic site level and $a_{ij}^{'}\sim N(0,{\varphi^{'}}^{2})$ is the random intercept at the individual level. $\epsilon_{ijk}^{'}\sim N(0,{\sigma^{'}}^{2})$ represents the error term. The coefficient $\beta_{9}^{'}$ is what we would like to evaluate whether it is statistically significantly different from zero.
