# Supplementary material for: Air quality improvement and cognitive decline in community-dwelling older women in the United States: A longitudinal cohort study
Source: PLoS Med. 2022 Feb 3;19(2):e1003893. doi: 10.1371/journal.pmed.1003893 (PMC8812844; doi:10.1371/journal.pmed.1003893)
Supplement: S7 Text — (DOCX) [file pmed.1003893.s008.docx]

**S7 Text.** **Time-Varying Propensity Score Approach to adjust for Selective Attrition due to Loss to Follow-Up**

We implemented a time-varying propensity score adjustment approach to try to control for possible differential attrition due to loss to follow-up in our study [1]. We calculated the probability of having the observed exposures over different follow-up intervals and included this periodically updated probability as a time-varying covariate in the linear mixed effect models to control for potential bias due to differential attrition.

The procedure included the following steps:

1) We divided the follow-up timeline into annual intervals so that women with cognitive function measured within the same year since WHIMS-ECHO enrollment were grouped together. We combined data after year 8 into one group, given the fewer women in these years.

2) We then constructed a linear regression model using air quality improvement as the outcome. For predictor variables, we included all baseline covariates (age at WHIMS-ECHO enrollment; race/ethnicity; geographic region; socioeconomic factors; lifestyle factors; clinical characteristics) in our model to determine which were significantly associated with air quality improvement. Our final model included race/ethnicity and geographic region as predictors and air quality improvement ($A_{t}$) as the dependent variable for each follow-up year *t* (t=0, 1, …, 8), as shown below:

$$A_{t}=\alpha+\beta_{1}*\mathrm{race}+\beta_{2}*region+\varepsilon, where \varepsilon\sim N\left( 0,\sigma^{2} \right)$$

Based on parameters ($\hat{\alpha},$ $\hat{\beta}_{1}$, $\hat{\beta}_{2},$and $\hat{\sigma}$) estimated from the above model, we calculated the probability of having the observed exposure $a_{i}$ for each individual *i* by the normal density [2]:

$f \left( a_{i} | {race}_{i},{region}_{i} \right)$ = $\frac{1}{\sqrt{2\pi\hat{\sigma}^{2}}}e^{-\left[ a_{i}-\left( \hat{\alpha}+\hat{\beta}_{1}{race}_{i}+\hat{\beta}_{2}{region}_{i} \right) \right]^{2}/2\hat{\sigma}^{2}}$

This is the preliminary propensity score (${PS}_{t}$) for each individual with a visit in year *t*.

3) The preliminary time-varying propensity score can be improved by considering covariate interactions in prediction models. However, we were unable to explicitly specify them due to too many possibilities. Instead, we improved propensity scores by adding an interaction term of the preliminary versions of an earlier and a later propensity score in the above linear regression model [1], as shown below:

$$A_{t}=\alpha^{'}+\beta_{1}^{'}*race+\beta_{2}^{'}*region+\beta_{3}^{'}{PS}_{t-1}{*PS}_{t+1}+\varepsilon^{'}, where \varepsilon^{'}\sim N\left( 0,{\sigma^{'}}^{2} \right)$$

The updated ${PS}_{t}'$ was then calculated as:

$\mathrm{PS}_{t}^{'}=f \left( a_{i} | {race}_{i},{region}_{i}, {PS}_{t-1},{PS}_{t+1} \right)$ = $\frac{1}{\sqrt{2\pi{\hat{\sigma^{'}}}^{2}}}e^{-\frac{\left[ a_{i}-\left( \hat{\alpha^{'}}+\hat{\beta_{1}^{'}}{race}_{i}+\hat{\beta_{2}^{'}}{region}_{i}+\hat{\beta_{3}^{'}}{PS}_{t-1}{*PS}_{t+1} \right) \right]^{2}}{2{\hat{\sigma^{'}}}^{2}}}$

Here, ${PS}_{t-1}$refers to a consecutive earlier preliminary propensity score and ${PS}_{t+1}$ refers to a consecutive later preliminary propensity score. It is important to note that these do not necessarily represent the preliminary propensity score values in the one year immediately prior to or after ${PS}_{t}$.

4) The final time-varying propensity score series were the updated versions of the propensity score (unless only the preliminary version was calculated) and these were then included in the models as a time-varying covariate to control for differential attrition in all analyses.

Supplemental References

1. Wyss R, Gagne JJ, Zhao Y, Zhou EH, Major JM, Wang SV, et al. Use of Time-Dependent Propensity Scores to Adjust Hazard Ratio Estimates in Cohort Studies with Differential Depletion of Susceptibles. Epidemiology. 2020;31(1):82-9. doi: 10.1097/EDE.0000000000001107. PubMed PMID: 31569120.

2. Robins JM, Hernán MA, Brumback B. Marginal structural models and causal inference in epidemiology. Epidemiology. 2000;11(5):550-60. doi: 10.1097/00001648-200009000-00011. PubMed PMID: 10955408.
