# Supplementary material for: Air quality improvement and cognitive decline in community-dwelling older women in the United States: A longitudinal cohort study
Source: PLoS Med. 2022 Feb 3;19(2):e1003893. doi: 10.1371/journal.pmed.1003893 (PMC8812844; doi:10.1371/journal.pmed.1003893)
Supplement: S8 Text — (DOCX) [file pmed.1003893.s009.docx]

**S8 Text. Multiple Imputation**

To evaluate the potential bias of conducting complete-case analyses, multiple imputation using fully conditional specification algorithm was used to handle missing data on air quality measures and key covariates [1,2]. Predictive mean matching or discriminant function methods were used to impute missing values for continuous or categorical variables, respectively. Air quality measures and all covariates used in the full multivariate linear mixed effect models were included in the imputation model, including demographics, individual- and neighborhood-level socioeconomic characteristics, lifestyle factors, and clinical characteristics. Imputation quality was evaluated by comparing the distributions before and after imputation and examining trace plots of the mean and standard deviation of each continuous variable in the imputation model. Linear mixed effect models using ten imputed data were re-run and effect estimates were summarized as comparison.

Supplemental References

1. Rubin DB. Multiple Imputation for Nonresponse in Surveys. New York: John Wiley & Sons Inc. 1987.

2. Van Buuren S, Brand JPL, Groothuis-Oudshoorn CGM, Rubin DB. Fully conditional specification in multivariate imputation. Journal of statistical computation and simulation. 2006;76(12):1049-64.
