# Supplementary material for: Air quality improvement and cognitive decline in community-dwelling older women in the United States: A longitudinal cohort study
Source: PLoS Med. 2022 Feb 3;19(2):e1003893. doi: 10.1371/journal.pmed.1003893 (PMC8812844; doi:10.1371/journal.pmed.1003893)
Supplement: S9 Text — (DOCX) [file pmed.1003893.s010.docx]

**S9 Text.** **Ascertainment of Probable Dementia**

Incident cases of probable dementia were determined using previously published WHIMS protocols [1]. Briefly, participants underwent an annual, validated telephone interview that comprised of a neuropsychological battery including the modified Telephone Interview for Cognitive Status (TICSm) and additional neuropsychological tests. If a woman scored below 31 on the TICSm, the standardized, validated Dementia Questionnaire was administered to a previously identified proxy (friend or family member). The results and the cognitive scoring history were then reviewed by a panel of experts in the diagnosis of dementia. Clinical diagnoses of dementia were decided by the central adjudication committee for final confirmation.

Supplemental References

1. Rapp SR, Legault C, Espeland MA, Resnick SM, Hogan PE, Coker LH, et al. Validation of a cognitive assessment battery administered over the telephone. J Am Geriatr Soc. 2012;60(9):1616-23. doi: 10.1111/j.1532-5415.2012.04111.x. PubMed PMID: 22985137; PubMed Central PMCID: PMCPMC3448122.
