# Supplementary figures and images for: Air quality improvement and cognitive decline in community-dwelling older women in the United States: A longitudinal cohort study
Source: PLoS Med. 2022 Feb 3;19(2):e1003893. doi: 10.1371/journal.pmed.1003893 (PMC8812844; doi:10.1371/journal.pmed.1003893)

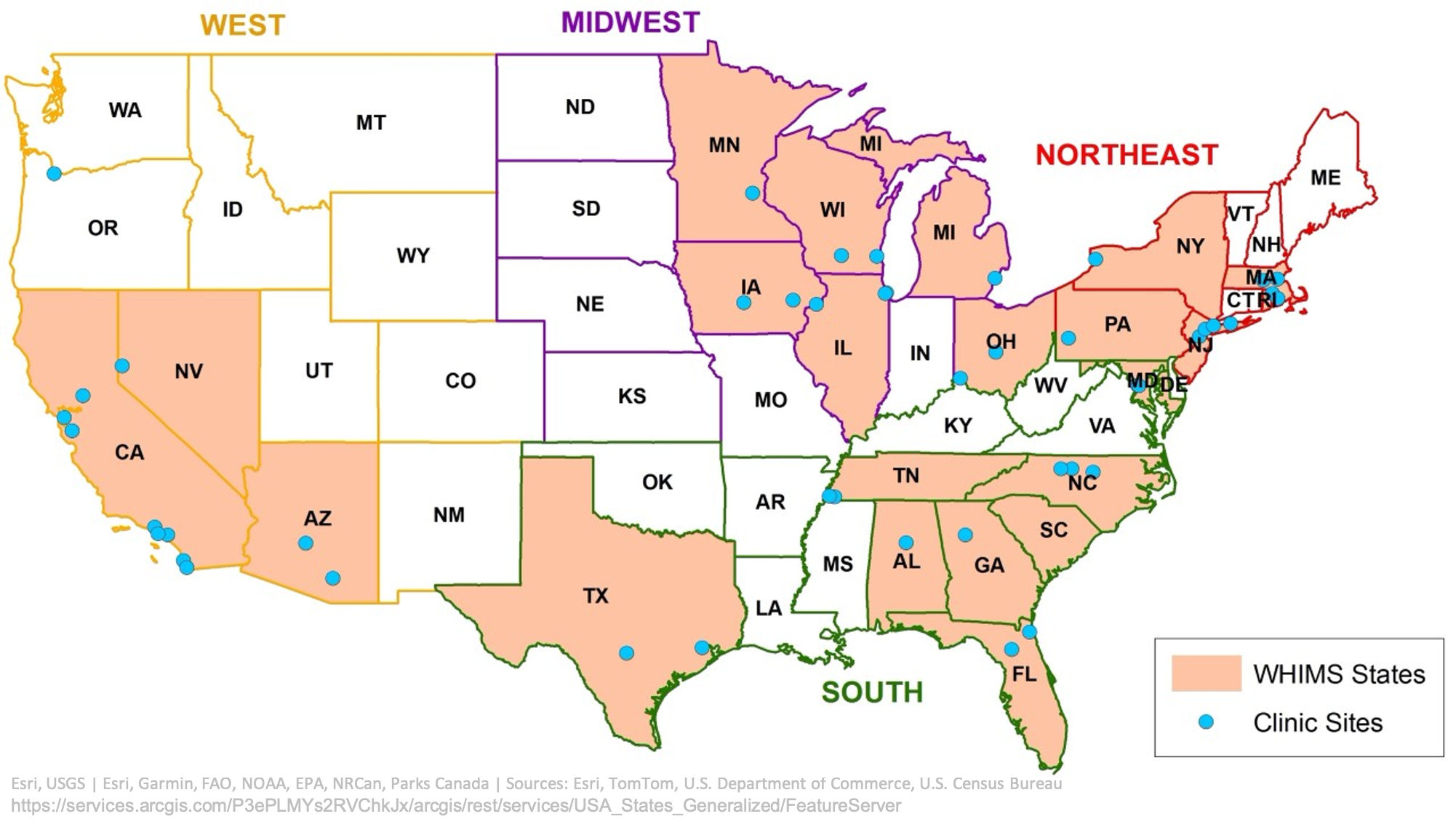

Supplement: S1 Fig — The direct link to the base layer of the map used in this figure: https://services.arcgis.com/P3ePLMYs2RVChkJx/arcgis/rest/services/USA_States_Generalized/FeatureServer. Maps were created using ArcGIS software by Esri. ArcGIS and ArcMap are the intellectual property of Esri and are used herein under license. Copyright Esri. All rights reserved. For more information about Esri software, please visit www.esri.com. WHIMS, Women’s Health Initiative Memory Study. (TIFF) [file pmed.1003893.s011.tiff]
