# Supplementary material for: Air quality improvement and cognitive decline in community-dwelling older women in the United States: A longitudinal cohort study
Source: PLoS Med. 2022 Feb 3;19(2):e1003893. doi: 10.1371/journal.pmed.1003893 (PMC8812844; doi:10.1371/journal.pmed.1003893)
Supplement: S1 Table — CVLT, California Verbal Learning Test. (DOCX) [file pmed.1003893.s012.docx]

**S1 Table. Comparing Study Samples with Repeated CVLT Measures vs. Excluded due to No Repeated CVLT Measures**

| **Characteristics** | **Study Sample for TICSm outcome (N=2534)^a^** | **Study Sample for CVLT outcome**  **(N=1947)^a^** | **Excluded**  **(N=587)^a^** | **p**^b^ |
| --- | --- | --- | --- | --- |
| **Region** |  |  |  | 0.45 |
| Northeast | 773 (30.5%) | 593 (30.5%) | 180 (30.7%) |  |
| South | 540 (21.3%) | 402 (20.6%) | 138 (23.5%) |  |
| Midwest | 618 (24.4%) | 482 (24.8%) | 136 (23.2%) |  |
| West | 603 (23.8%) | 470 (24.1%) | 133 (22.7%) |  |
| **Age** |  |  |  | <0.001 |
| ≤ 80 years | 979 (38.6%) | 808 (41.5%) | 171 (29.1%) |  |
| > 80 years | 1555 (61.4%) | 1139 (58.5%) | 416 (70.9%) |  |
| **Ethnicity** |  |  |  | 0.82 |
| Black (not Hispanic) | 160 (6.3%) | 120 (6.2%) | 40 (6.8%) |  |
| White (not Hispanic) | 2262 (89.3%) | 1742 (89.5%) | 520 (88.6%) |  |
| Other or Missing | 112 (4.4%) | 85 (4.4%) | 27 (4.6%) |  |
| **Education** |  |  |  | <0.001 |
| ≤ High school or GED | 660 (26.1%) | 476 (24.5%) | 184 (31.3%) |  |
| > High school but < 4y of college | 975 (38.5%) | 733 (37.7%) | 242 (41.2%) |  |
| ≥ 4y of college | 895 (35.4%) | 734 (37.8%) | 161 (27.4%) |  |
| **Employment** |  |  |  | 0.18 |
| Currently working | 388 (15.4%) | 310 (16.0%) | 78 (13.4%) |  |
| Not working | 238 (9.4%) | 188 (9.7%) | 50 (8.6%) |  |
| Retired | 1899 (75.2%) | 1443 (74.3%) | 456 (78.1%) |  |
| **Income ($)** |  |  |  | <0.001 |
| < 9,999 | 94 (3.7%) | 67 (3.4%) | 27 (4.6%) |  |
| 10,000-34,999 | 1140 (45%) | 849 (43.6%) | 291 (49.6%) |  |
| 35,000-74,999 | 927 (36.6%) | 741 (38.1%) | 186 (31.7%) |  |
| 75,000 or more | 242 (9.6%) | 204 (10.5%) | 38 (6.5%) |  |
| Do not know | 131 (5.2%) | 86 (4.4%) | 45 (7.7%) |  |
| **Smoking status** |  |  |  | 0.96 |
| Never smoked | 1396 (55.6%) | 1074 (55.7%) | 322 (55.1%) |  |
| Past smoker | 996 (39.6%) | 763 (39.6%) | 233 (39.9%) |  |
| Current Smoker | 121 (4.8%) | 92 (4.8%) | 29 (5.0%) |  |
| **Alcohol use** |  |  |  | 0.01 |
| Non-drinker | 307 (12.2%) | 227 (11.7%) | 80 (13.7%) |  |
| Past drinker | 436 (17.3%) | 313 (16.2%) | 123 (21.1%) |  |
| < 1 drink per day | 1466 (58.2%) | 1150 (59.4%) | 316 (54.3%) |  |
| ≥ 1 drink per day | 310 (12.3%) | 247 (12.8%) | 63 (10.8%) |  |
| **Moderate or strenuous physical activities ≥ 20 minutes** |  |  |  | 0.31 |
| No activity | 1387 (54.8%) | 1053 (54.1%) | 334 (56.9%) |  |
| Some activity | 138 (5.5%) | 102 (5.2%) | 36 (6.1%) |  |
| 2-4 episodes/week | 534 (21.1%) | 413 (21.2%) | 121 (20.6%) |  |
| > 4 episodes/week | 473 (18.7%) | 377 (19.4%) | 96 (16.4%) |  |
| **Body Mass Index (kg/m^2^)** |  |  |  | 0.98 |
| < 25 | 701 (27.8%) | 538 (27.7%) | 163 (28%) |  |
| 25-29 | 931 (36.9%) | 718 (37.0%) | 213 (36.5%) |  |
| ≥ 30 | 890 (35.3%) | 683 (35.2%) | 207 (35.5%) |  |
| **Hypertension** |  |  |  | 0.02 |
| No | 1646 (65.5%) | 1289 (66.7%) | 357 (61.3%) |  |
| Yes | 868 (34.5%) | 643 (33.3%) | 225 (38.7%) |  |
| **Hypercholesterolemia** |  |  |  | 0.28 |
| No | 2063 (82.6%) | 1595 (83.1%) | 468 (81.1%) |  |
| Yes | 434 (17.4%) | 325 (16.9%) | 109 (18.9%) |  |
| **Diabetes** |  |  |  | 0.19 |
| No | 2433 (96.1%) | 1876 (96.4%) | 557 (95.2%) |  |
| Yes | 98 (3.9%) | 70 (3.6%) | 28 (4.8%) |  |
| **Cardiovascular disease history** |  |  |  | 0.40 |
| No | 2143 (85.7%) | 1652 (86.0%) | 491 (84.7%) |  |
| Yes | 357 (14.3%) | 268 (14.0%) | 89 (15.3%) |  |
| **Any prior postmenopausal hormone treatment** |  |  |  | 0.79 |
| No | 1373 (54.2%) | 1052 (54.1%) | 321 (54.7%) |  |
| Yes | 1160 (45.8%) | 894 (45.9%) | 266 (45.3%) |  |
| **WHI Hormone Therapy Assignment** |  |  |  | 0.38 |
| CEE-alone placebo | 459 (18.1%) | 346 (17.8%) | 113 (19.3%) |  |
| CEE-alone | 461 (18.2%) | 345 (17.7%) | 116 (19.8%) |  |
| CEE+MPA placebo | 832 (32.8%) | 654 (33.6%) | 178 (30.3%) |  |
| CEE+MPA | 782 (30.9%) | 602 (30.9%) | 180 (30.7%) |  |
| **ApoE**^c^ |  |  |  | <0.001 |
| e2/2+e2/3+e3/3 | 1382 (77.5%) | 1097 (79.3%) | 285 (71.4%) |  |
| e2/4+e3/4+e4/4 | 401 (22.5%) | 287 (20.7%) | 114 (28.6%) |  |

Abbreviations: TICSm, modified Telephone Interview for Cognitive Status; CVLT, California Verbal Learning Tests; GED, general educational development; WHI: Women’s Health Initiative; CEE, conjugated equine estrogens; MPA, medroxyprogesterone acetate; ApoE: Apolipoprotein E

^a^ Numbers in the samples may not added up to total due to missing.

^b^ P Values were calculated using chi-square tests.

^c^ Numbers in the samples with ApoE genotyping did not add up to the total due to missing.
