# Supplementary material for: Air quality improvement and cognitive decline in community-dwelling older women in the United States: A longitudinal cohort study
Source: PLoS Med. 2022 Feb 3;19(2):e1003893. doi: 10.1371/journal.pmed.1003893 (PMC8812844; doi:10.1371/journal.pmed.1003893)
Supplement: S2 Table — AQ, air quality. (DOCX) [file pmed.1003893.s013.docx]

**S2 Table. Pearson Correlations^a^ between Air Quality Measures**

| **A) In analytic sample for TICSm outcome (N=2232)** | | | | | | | | |
| --- | --- | --- | --- | --- | --- | --- | --- | --- |
|  |  | **PM_2.5_ (µg/m^3^)**^b^ | | |  | **NO_2_ (ppb)**^b^ | | |
|  |  | **Air quality improvement** | **Recent exposure** | **Remote exposure** |  | **Air quality improvement** | **Recent exposure** | **Remote exposure** |
| **PM_2.5_** | **Air quality improvement** | 1 |  |  |  |  |  |  |
|  | **Recent exposure** | 0.09 | 1 |  |  |  |  |  |
|  | **Remote exposure** | 0.67 | 0.80 | 1 |  |  |  |  |
| **NO_2_** | **Air quality improvement** | 0.61 | 0.19 | 0.51 |  | 1 |  |  |
|  | **Recent exposure** | 0.34 | 0.48 | 0.56 |  | 0.45 | 1 |  |
|  | **Remote exposure** | 0.53 | 0.42 | 0.63 |  | 0.79 | 0.90 | 1 |
| **B) In analytic sample for CVLT outcome (N=1721)** | | | | | | | | |
|  |  | **PM_2.5_ (µg/m^3^)**^b^ | | |  | **NO_2_ (ppb)**^b^ | | |
|  |  | **Air quality improvement** | **Recent exposure** | **Remote exposure** |  | **Air quality improvement** | **Recent exposure** | **Remote exposure** |
| **PM_2.5_** | **Air quality improvement** | 1 |  |  |  |  |  |  |
|  | **Recent exposure** | 0.10 | 1 |  |  |  |  |  |
|  | **Remote exposure** | 0.69 | 0.79 | 1 |  |  |  |  |
| **NO_2_** | **Air quality improvement** | 0.63 | 0.19 | 0.53 |  | 1 |  |  |
|  | **Recent exposure** | 0.35 | 0.50 | 0.58 |  | 0.45 | 1 |  |
|  | **Remote exposure** | 0.55 | 0.43 | 0.65 |  | 0.79 | 0.90 | 1 |

Abbreviations: TICSm, modified Telephone Interview for Cognitive Status; CVLT, California Verbal Learning Tests; PM_2.5_, fine particulate matter; NO_2_, nitrogen dioxide

^a^ P Values were calculated using t-tests. All p-values < 0.001.

^b^ Recent exposures were the 3-year average exposures estimated at the WHIMS-ECHO enrollment. Remote exposures were the 3-year average exposures estimated 10 years before the WHIMS-ECHO enrollment. Air quality improvement was defined as reduction from the remote to recent exposures over the 10-year period.
