# Supplementary material for: Air quality improvement and cognitive decline in community-dwelling older women in the United States: A longitudinal cohort study
Source: PLoS Med. 2022 Feb 3;19(2):e1003893. doi: 10.1371/journal.pmed.1003893 (PMC8812844; doi:10.1371/journal.pmed.1003893)
Supplement: S3 Table — (DOCX) [file pmed.1003893.s014.docx]

**S3 Table. Distribution of Cognitive Test Scores by Visit**

|  | **TICSm score** | |  | **CVLT score** | |
| --- | --- | --- | --- | --- | --- |
| **Visit** | **n** | **Mean ± SD** |  | **n** | **Mean ± SD** |
| 1 | 2232 | 35.08 ± 4.62 |  | 1721 | 24.52 ± 7.12 |
| 2 | 2232 | 34.80 ± 5.20 |  | 1721 | 24.21 ± 7.65 |
| 3 | 1975 | 34.92 ± 5.12 |  | 1505 | 23.93 ± 7.84 |
| 4 | 1751 | 34.81 ± 5.28 |  | 1310 | 24.09 ± 8.00 |
| 5 | 1545 | 34.58 ± 5.29 |  | 1081 | 24.27 ± 7.90 |
| 6 | 1357 | 34.22 ± 5.42 |  | 885 | 23.78 ± 8.03 |
| 7 | 1137 | 34.38 ± 4.80 |  | 646 | 24.28 ± 7.66 |
| 8 | 933 | 34.42 ± 4.99 |  | 158 | 25.18 ± 7.54 |
| 9 | 691 | 34.32 ± 4.44 |  |  |  |
| 10 | 374 | 34.88 ± 3.92 |  |  |  |

Abbreviations: TICSm, modified Telephone Interview for Cognitive Status; CVLT, California Verbal Learning Tests; SD: standard deviation
