# Supplementary material for: Air quality improvement and cognitive decline in community-dwelling older women in the United States: A longitudinal cohort study
Source: PLoS Med. 2022 Feb 3;19(2):e1003893. doi: 10.1371/journal.pmed.1003893 (PMC8812844; doi:10.1371/journal.pmed.1003893)
Supplement: S4 Table — (DOCX) [file pmed.1003893.s015.docx]

**S4 Table. Distribution of Cognitive Scores at Baseline and Last Visits by Population Characteristics**

|  | **Analytic sample for TICSm outcome** | | | | | **Analytic sample for CVLT outcome** | | | | |
| --- | --- | --- | --- | --- | --- | --- | --- | --- | --- | --- |
|  |  | **base TICSm score** | | **last TICSm score^a^** | |  | **base CVLT score** | | **last CVLT score^a^** | |
| **Population Characteristics** | **N** | **Mean ± SD** | **P^b^** | **Mean ± SD** | **P^b^** | **N** | **Mean ± SD** | **P^b^** | **Mean ± SD** | **P^b^** |
| Overall | 2332 | 35.08 ± 4.62 |  | 31.87 ± 6.19 |  | 1721 | 24.52 ± 7.12 |  | 21.15 ± 8.55 |  |
| Region |  |  | 0.02 |  | 0.19 |  |  | <0.001 |  | 0.09 |
| Northeast | 718 | 35.01 ± 4.58 |  | 31.63 ± 6.38 |  | 551 | 23.68 ± 6.95 |  | 20.50 ± 8.88 |  |
| South | 443 | 34.69 ± 4.74 |  | 31.78 ± 6.52 |  | 329 | 23.59 ± 6.94 |  | 21.12 ± 8.06 |  |
| Midwest | 549 | 35.59 ± 4.37 |  | 32.36 ± 5.70 |  | 428 | 25.55 ± 6.97 |  | 21.90 ± 8.47 |  |
| West | 522 | 35.00 ± 4.80 |  | 31.78 ± 6.14 |  | 413 | 25.33 ± 7.40 |  | 21.28 ± 8.53 |  |
| Age |  |  | <0.001 |  | <0.001 |  |  | <0.001 |  | <0.001 |
| ≤ 80 years | 879 | 36.10 ± 4.38 |  | 33.28 ± 5.99 |  | 731 | 25.96 ± 6.74 |  | 22.94 ± 8.54 |  |
| > 80 years | 1353 | 34.42 ± 4.66 |  | 30.96 ± 6.16 |  | 990 | 23.46 ± 7.20 |  | 19.83 ± 8.32 |  |
| Ethnicity |  |  | <0.001 |  | <0.001 |  |  | 0.03 |  | 0.37 |
| Black (not Hispanic) | 116 | 32.96 ± 5.30 |  | 29.44 ± 7.24 |  | 86 | 22.58 ± 7.26 |  | 19.91 ± 8.51 |  |
| White (not Hispanic) | 2042 | 35.22 ± 4.53 |  | 32.05 ± 6.11 |  | 1576 | 24.63 ± 7.09 |  | 21.23 ± 8.57 |  |
| Other | 74 | 34.72 ± 5.24 |  | 30.92 ± 6.02 |  | 59 | 24.49 ± 7.30 |  | 20.98 ± 8.23 |  |
| Education |  |  | <0.001 |  | <0.001 |  |  | 0.002 |  | 0.07 |
| ≤ High school or GED | 564 | 33.90 ± 4.94 |  | 31.12 ± 6.31 |  | 406 | 23.41 ± 7.19 |  | 20.31 ± 8.46 |  |
| > High school but < 4y of college | 864 | 35.00 ± 4.58 |  | 31.78 ± 6.01 |  | 651 | 24.84 ± 6.87 |  | 21.52 ± 8.21 |  |
| ≥ 4y of college | 804 | 36.00 ± 4.23 |  | 32.50 ± 6.25 |  | 664 | 24.89 ± 7.25 |  | 21.31 ± 8.91 |  |
| Employment |  |  | 0.01 |  | <0.001 |  |  | 0.02 |  | 0.003 |
| Currently working | 348 | 35.69 ± 4.68 |  | 33.16 ± 5.53 |  | 279 | 25.51 ± 7.03 |  | 22.70 ± 8.51 |  |
| Not working | 210 | 34.60 ± 4.26 |  | 31.38 ± 6.23 |  | 166 | 23.75 ± 7.69 |  | 20.32 ± 8.54 |  |
| Retired | 1674 | 35.02 ± 4.64 |  | 31.67 ± 6.29 |  | 1276 | 24.41 ± 7.04 |  | 20.92 ± 8.53 |  |
| Income ($) |  |  | <0.001 |  | <0.001 |  |  | 0.03 |  | 0.01 |
| < 9,999 | 74 | 32.72 ± 5.90 |  | 29.20 ± 7.42 |  | 52 | 22.62 ± 7.13 |  | 18.90 ± 8.00 |  |
| 10,000-34,999 | 1000 | 34.58 ± 4.70 |  | 31.48 ± 6.02 |  | 742 | 24.17 ± 6.93 |  | 20.97 ± 8.40 |  |
| 35,000-74,999 | 824 | 35.71 ± 4.32 |  | 32.37 ± 6.13 |  | 663 | 24.90 ± 7.14 |  | 21.24 ± 8.55 |  |
| 75,000 or more | 220 | 35.89 ± 4.15 |  | 33.23 ± 5.81 |  | 186 | 25.39 ± 7.43 |  | 22.86 ± 9.05 |  |
| Don't know | 114 | 34.98 ± 4.94 |  | 30.85 ± 7.10 |  | 78 | 23.82 ± 7.55 |  | 19.60 ± 8.63 |  |
| Lifestyle |  |  |  |  |  |  |  |  |  |  |
| Smoking status |  |  | 0.94 |  | 0.46 |  |  | 0.02 |  | 0.17 |
| Never smoked | 1239 | 35.05 ± 4.73 |  | 31.97 ± 6.35 |  | 954 | 24.80 ± 7.06 |  | 21.30 ± 8.49 |  |
| Past smoker | 890 | 35.12 ± 4.44 |  | 31.70 ± 5.98 |  | 689 | 24.00 ± 7.22 |  | 20.80 ± 8.68 |  |
| Current Smoker | 103 | 35.12 ± 4.89 |  | 32.32 ± 6.12 |  | 78 | 25.73 ± 6.59 |  | 22.55 ± 7.98 |  |
| Alcohol use |  |  | <0.001 |  | 0.002 |  |  | 0.002 |  | 0.06 |
| Non-drinker | 261 | 34.38 ± 4.85 |  | 31.07 ± 6.89 |  | 191 | 23.95 ± 7.08 |  | 20.38 ± 8.34 |  |
| Past drinker | 372 | 34.22 ± 5.12 |  | 31.18 ± 6.53 |  | 266 | 23.23 ± 7.13 |  | 20.32 ± 7.79 |  |
| < 1 drink per day | 1315 | 35.32 ± 4.46 |  | 32.06 ± 5.94 |  | 1036 | 24.75 ± 7.07 |  | 21.29 ± 8.68 |  |
| ≥ 1 drink per day | 284 | 35.76 ± 4.22 |  | 32.67 ± 6.11 |  | 228 | 25.46 ± 7.14 |  | 22.14 ± 8.87 |  |
| Moderate or strenuous physical activities ≥ 20 minutes |  |  | 0.06 |  | 0.40 |  |  | 0.71 |  | 0.47 |
| No activity | 1207 | 34.91 ± 4.72 |  | 32.04 ± 6.04 |  | 917 | 24.38 ± 6.88 |  | 21.24 ± 8.45 |  |
| Some activity | 124 | 34.69 ± 4.72 |  | 31.81 ± 6.63 |  | 91 | 24.49 ± 7.12 |  | 21.91 ± 8.55 |  |
| 2-4 episodes/week | 481 | 35.22 ± 4.45 |  | 31.47 ± 6.38 |  | 376 | 24.52 ± 7.37 |  | 20.60 ± 8.68 |  |
| > 4 episodes/week | 420 | 35.55 ± 4.47 |  | 31.89 ± 6.29 |  | 337 | 24.91 ± 7.47 |  | 21.33 ± 8.67 |  |
| Physical Health |  |  |  |  |  |  |  |  |  |  |
| Body Mass Index (kg/m^2^) |  |  | 0.53 |  | 0.76 |  |  | 0.95 |  | 0.71 |
| < 25 | 619 | 35.13 ± 4.55 |  | 31.76 ± 6.44 |  | 476 | 24.48 ± 7.20 |  | 21.13 ± 8.76 |  |
| 25-29 | 815 | 35.19 ± 4.56 |  | 32.00 ± 5.88 |  | 633 | 24.49 ± 6.91 |  | 21.36 ± 8.35 |  |
| ≥ 30 | 798 | 34.94 ± 4.74 |  | 31.83 ± 6.31 |  | 612 | 24.60 ± 7.27 |  | 20.96 ± 8.60 |  |
| Hypertension |  |  | <0.001 |  | 0.12 |  |  | 0.002 |  | 0.01 |
| No | 1461 | 35.33 ± 4.58 |  | 32.02 ± 6.18 |  | 1152 | 24.89 ± 7.01 |  | 21.51 ± 8.54 |  |
| Yes | 771 | 34.61 ± 4.67 |  | 31.59 ± 6.22 |  | 569 | 23.77 ± 7.28 |  | 20.43 ± 8.53 |  |
| Hypercholesterolemia |  |  | 0.08 |  | 0.41 |  |  | 0.36 |  | 0.99 |
| No | 1855 | 35.16 ± 4.58 |  | 31.92 ± 6.15 |  | 1438 | 24.59 ± 7.08 |  | 21.15 ± 8.58 |  |
| Yes | 377 | 34.71 ± 4.81 |  | 31.64 ± 6.43 |  | 283 | 24.17 ± 7.31 |  | 21.16 ± 8.43 |  |
| Diabetes |  |  | 0.17 |  | 0.24 |  |  | 0.04 |  | 0.34 |
| No | 2143 | 35.11 ± 4.62 |  | 31.91 ± 6.19 |  | 1655 | 24.59 ± 7.08 |  | 21.19 ± 8.58 |  |
| Yes | 89 | 34.43 ± 4.74 |  | 31.12 ± 6.37 |  | 66 | 22.77 ± 7.72 |  | 20.17 ± 7.77 |  |
| Cardiovascular disease history |  |  | 0.03 |  | 0.07 |  |  | 0.16 |  | 0.33 |
| No | 1907 | 35.17 ± 4.61 |  | 31.97 ± 6.24 |  | 1476 | 24.62 ± 7.21 |  | 21.24 ± 8.66 |  |
| Yes | 325 | 34.56 ± 4.69 |  | 31.30 ± 5.92 |  | 245 | 23.94 ± 6.53 |  | 20.66 ± 7.84 |  |
| Any prior hormone therapy |  |  | 0.01 |  | 0.03 |  |  | 0.46 |  | 0.83 |
| No | 1218 | 35.30 ± 4.63 |  | 32.14 ± 6.10 |  | 935 | 24.64 ± 6.98 |  | 21.19 ± 8.76 |  |
| Yes | 1014 | 34.82 ± 4.60 |  | 31.56 ± 6.29 |  | 786 | 24.38 ± 7.27 |  | 21.11 ± 8.31 |  |
| WHI Therapy Assignment |  |  | 0.03 |  | 0.19 |  |  | 0.81 |  | 0.93 |
| CEE-alone placebo | 400 | 35.16 ± 4.86 |  | 31.88 ± 5.93 |  | 306 | 24.59 ± 7.35 |  | 21.42 ± 8.57 |  |
| CEE-alone | 403 | 34.49 ± 4.75 |  | 31.28 ± 6.51 |  | 302 | 24.28 ± 7.07 |  | 20.96 ± 8.03 |  |
| CEE+ MPA placebo**s** | 733 | 35.30 ± 4.39 |  | 32.01 ± 6.06 |  | 578 | 24.72 ± 6.90 |  | 21.17 ± 8.60 |  |
| CEE+MPA | 696 | 35.16 ± 4.63 |  | 32.08 ± 6.29 |  | 535 | 24.41 ± 7.25 |  | 21.09 ± 8.79 |  |
| ApoE^c^ |  |  | <0.001 |  | <0.001 |  |  | 0.001 |  | <0.001 |
| e2/2+e2/3+e3/3 | 1239 | 35.59 ± 4.43 |  | 32.50 ± 5.97 |  | 984 | 24.83 ± 6.94 |  | 21.76 ± 8.47 |  |
| e2/4+e3/4+e4/4 | 372 | 34.20 ± 4.87 |  | 30.54 ± 6.34 |  | 269 | 23.26 ± 7.61 |  | 19.20 ± 8.73 |  |

Abbreviations: TICSm, modified Telephone Interview for Cognitive Status; CVLT, California Verbal Learning Tests; SD: standard deviation; GED, general educational development; WHI: Women’s Health Initiative; CEE, conjugated equine estrogens; MPA, medroxyprogesterone acetate; ApoE: Apolipoprotein E

^a^ The visit time of the last test may differ due to attrition.

^b^ P values were calculated using ANOVA F-tests for mean exposures.

^c^ Numbers in the samples with ApoE genotyping did not add up to the total due to missing.
