# Supplementary material for: Air quality improvement and cognitive decline in community-dwelling older women in the United States: A longitudinal cohort study
Source: PLoS Med. 2022 Feb 3;19(2):e1003893. doi: 10.1371/journal.pmed.1003893 (PMC8812844; doi:10.1371/journal.pmed.1003893)
Supplement: S5 Table — AQ, air quality; WHI, Women’s Health Initiative; WHIMS-ECHO, Women’s Health Initiative Memory Study-Epidemiology of Cognitive Health Outcomes. (DOCX) [file pmed.1003893.s016.docx]

**S5 Table. Summary of Sensitivity Analyses for the Associations between Air Quality Improvement and Cognitive Decline, with Adjustment of Covariates Assessed at the WHIMS-ECHO Enrollment or Changes from WHI Inception to WHIMS-ECHO Enrollment**

| **A) Associations with declines in general cognitive status (N=2232)** | | | | | | | |
| --- | --- | --- | --- | --- | --- | --- | --- |
|  | **Sensitivity Analysis** | **Air quality improvement in PM_2.5_^a^** | | | **Air quality improvement in NO_2_^a^** | | |
| **Models** | **Scenarios** | **β^b^** | **95% CI** | **p^c^** | **β^b^** | **95% CI** | **p^c^** |
| **Model I ^d^** | Adjusted for covariates assessed at the WHIMS-ECHO enrollment | 0.025 | 0.0004, 0.05 | 0.046 | 0.034 | 0.01, 0.06 | 0.006 |
| **Model II ^e^** | Adjusted for changes from WHI inception to WHIMS-ECHO enrollment | 0.025 | 0.0005, 0.05 | 0.045 | 0.034 | 0.01, 0.06 | 0.006 |
| **B) Associations with declines in episodic memory (N=1721)** | | | | | | | |
|  | **Sensitivity Analysis** | **Air quality improvement in PM_2.5_^a^** | | | **Air quality improvement in NO_2_^a^** | | |
| **Models** | **Scenarios** | **β^b^** | **95% CI** | **p^c^** | **β^b^** | **95% CI** | **p^c^** |
| **Model I ^d^** | Adjusted for covariates assessed at the WHIMS-ECHO enrollment | 0.071 | 0.02, 0.12 | 0.009 | 0.061 | 0.01, 0.12 | 0.03 |
| **Model II ^e^** | Adjusted for changes from WHI inception to WHIMS-ECHO enrollment | 0.071 | 0.02, 0.12 | 0.01 | 0.061 | 0.01, 0.12 | 0.03 |

Abbreviations: WHI-CT (HT), Women’s Health Initiative-Clinical Trial (Hormone Therapy); WHIMS-ECHO, Women’s Health Initiative Memory Study-Epidemiology of Cognitive Health Outcomes; TICSm, modified Telephone Interview for Cognitive Status; CVLT, California Verbal Learning Tests; PM_2.5_, fine particulate matter; NO_2_, nitrogen dioxide

^a^ Recent exposures were the 3-year average exposures estimated at the WHIMS-ECHO enrollment. Remote exposures were the 3-year average exposures estimated 10 years before the WHIMS-ECHO enrollment. Air quality improvement was defined as reduction from the remote to recent exposures over the 10-year period.

^b^ β (95% CI) = regression coefficient (95% confidence interval) estimating the increase in TICSm score or CVLT score per year for each interquartile range (IQR) increase of air quality improvement (IQR_PM2.5_ = 1.79 µg/m^3^ for both TICSm and CVLT analytic samples; IQR_NO2_ = 3.92 ppb for TICSm analytic sample and 3.97 ppb for CVLT analytic sample). Positive coefficients represent slower decline associated with greater air quality improvement.

^c^ P values were calculated using Wald t-tests.

^d^ Model I: adjusted for spatial random effect, WHIMS-ECHO enrollment year, age, follow-up year, age interaction with follow-up year, demographic variables (geographic region and race/ethnicity), socioeconomic factors (education, income, employment status) and neighborhood socioeconomic characteristics, lifestyle factors (smoking, drinking and physical activities), prior hormone use, hormone therapy assignment, cardiovascular risk factors (hypertension, diabetes and hypercholesterolemia), depression, body mass index (BMI), cardiovascular disease (CVD) histories, and time-varying propensity scores. Here neighborhood socioeconomic characteristics, lifestyle factors (smoking, drinking and physical activities), hypertension, BMI, and CVD histories were assessed at the WHIMS-ECHO enrollment, instead of those assessed at the WHI inception.

^e^ Model II: adjusted for spatial random effect, WHIMS-ECHO enrollment year, age, follow-up year, age interaction with follow-up year, demographic variables (geographic region and race/ethnicity), socioeconomic factors (education, income, employment status) and neighborhood socioeconomic characteristics, lifestyle factors (smoking, drinking and physical activities), prior hormone use, hormone therapy assignment, cardiovascular risk factors (hypertension, diabetes and hypercholesterolemia), depression, BMI, CVD histories, time-varying propensity scores and changes from WHI inception to WHIMS-ECHO enrollment for the neighborhood socioeconomic characteristics, lifestyle factors (smoking, drinking and physical activities), hypertension, BMI, and CVD histories.
