# Supplementary material for: Air quality improvement and cognitive decline in community-dwelling older women in the United States: A longitudinal cohort study
Source: PLoS Med. 2022 Feb 3;19(2):e1003893. doi: 10.1371/journal.pmed.1003893 (PMC8812844; doi:10.1371/journal.pmed.1003893)
Supplement: S6 Table — AQ, air quality. (DOCX) [file pmed.1003893.s017.docx]

**S6 Table. Summary of the Associations between Air Quality Improvements and Cognitive Decline, with Missing Air Quality Measures and Covariates Imputed using Multiple Imputation^a^**

| **Cognitive** | **Sample** | **Air quality improvement in PM_2.5_^b^** | | | **Air quality improvement in NO_2_^b^** | | |
| --- | --- | --- | --- | --- | --- | --- | --- |
| **Outcome** | **Size** | **β^c^** | **95% CI** | **p^d^** | **β^c^** | **95% CI** | **p^d^** |
| **TICSm** | 2534 | 0.027 | 0.003, 0.05 | 0.02 | 0.029 | 0.006, 0.05 | 0.01 |
| **CVLT** | 1947 | 0.064 | 0.01, 0.11 | 0.01 | 0.053 | 0.0006, 0.11 | 0.047 |

Abbreviations: WHIMS-ECHO, Women’s Health Initiative Memory Study-Epidemiology of Cognitive Health Outcomes; TICSm, modified Telephone Interview for Cognitive Status; CVLT, California Verbal Learning Tests; PM_2.5_, fine particulate matter; NO_2_, nitrogen dioxide

**^a^** Missing data in air quality measures and covariates were imputed using multiple imputation. The β and 95% confidence intervals (95% CI) were computed based on linear mixed effect modeling results from 10 imputations.

**^b^** Recent exposures were the 3-year average exposures estimated at the WHIMS-ECHO enrollment. Remote exposures were the 3-year average exposures estimated 10 years before the WHIMS-ECHO enrollment. Air quality improvement was defined as reduction from the remote to recent exposures over the 10-year period.

**^c^** β = regression coefficient that estimates the increase in TICSm score or CVLT score per year for each interquartile range (IQR) increase of air quality improvement (IQR_PM2.5_ = 1.79 µg/m^3^ for both analytic samples; IQR_NO2_ = 3.92 ppb for TICSm analytic sample and 3.97 ppb for CVLT analytic sample), adjusting for spatial random effect, WHIMS-ECHO enrollment year, age, follow-up year, age interaction with follow-up year, time-varying propensity scores, demographic variables (geographic region and race/ethnicity), socioeconomic factors (education, income, employment status) and neighborhood socioeconomic characteristics, lifestyle factors (smoking, drinking and physical activities), prior hormone use, hormone therapy assignment, cardiovascular risk factors (hypertension, diabetes and hypercholesterolemia), depression, body mass index, and cardiovascular disease histories. Positive coefficients represent slower decline associated with greater air quality improvement.

^d^ P values were calculated using Wald t-tests.
