# Supplementary material for: Air quality improvement and cognitive decline in community-dwelling older women in the United States: A longitudinal cohort study
Source: PLoS Med. 2022 Feb 3;19(2):e1003893. doi: 10.1371/journal.pmed.1003893 (PMC8812844; doi:10.1371/journal.pmed.1003893)
Supplement: S7 Table — AQ, air quality. (DOCX) [file pmed.1003893.s018.docx]

**S7 Table. Summary of the Associations between Air Quality Measures and Cognitive Decline, with Single Exposure or Multiple Exposures in One Model**

| **A) Associations with declines in general cognitive status (N=2232)** | | | | | | | | | |
| --- | --- | --- | --- | --- | --- | --- | --- | --- | --- |
| **Models** | **Air quality improvement in** **PM_2.5_^a^** | | | **Recent PM_2.5_ exposure^a^** | | | **Remote PM_2.5_ exposure^a^** | | |
|  | **β^b^** | **95% CI** | **p^c^** | **β^b^** | **95% CI** | **p^c^** | **β^b^** | **95% CI** | **p^c^** |
| **Model I** | 0.026 | 0.001, 0.05 | 0.04 | **-** | **-** | **-** | **-** | **-** | **-** |
| **Model II** | **-** | **-** | **-** | 0.010 | -0.02, 0.04 | 0.52 | **-** | **-** | **-** |
| **Model III** | **-** | **-** | **-** | **-** | **-** | **-** | 0.023 | -0.004, 0.05 | 0.09 |
| **Model IV^d^** | 0.026 | 0.001, 0.05 | 0.04 | 0.008 | -0.02, 0.04 | 0.61 | **-** | **-** | **-** |
| **Model V^e^** | 0.021 | -0.01, 0.05 | 0.20 | **-** | **-** | **-** | 0.009 | -0.03, 0.04 | 0.61 |
| **Models** | **Air quality improvement in** **NO_2_^a^** | | | **Recent NO_2_ exposure^a^** | | | **Remote NO_2_ exposure^a^** | | |
|  | **β^b^** | **95% CI** | **p^c^** | **β^b^** | **95% CI** | **p^c^** | **β^b^** | **95% CI** | **p^c^** |
| **Model I** | 0.034 | 0.01, 0.06 | 0.005 | **-** | **-** | **-** | **-** | **-** | **-** |
| **Model II** | **-** | **-** | **-** | -0.001 | -0.03, 0.03 | 0.93 | **-** | **-** | **-** |
| **Model III** | **-** | **-** | **-** | **-** | **-** | **-** | 0.018 | -0.01, 0.05 | 0.23 |
| **Model IV^d^** | 0.041 | 0.01, 0.07 | 0.002 | -0.019 | -0.05, 0.01 | 0.21 | **-** | **-** | **-** |
| **Model V^e^** | 0.054 | 0.02, 0.09 | 0.006 | **-** | **-** | **-** | -0.030 | -0.08, 0.02 | 0.21 |
| **B) Associations with declines in episodic memory (N=1721)** | | | | | | | | | |
| **Models** | **Air quality improvement in** **PM_2.5_^a^** | | | **Recent PM_2.5_ exposure^a^** | | | **Remote PM_2.5_ exposure^a^** | | |
|  | **β^b^** | **95% CI** | **p^c^** | **β^b^** | **95% CI** | **p^c^** | **β^b^** | **95% CI** | **p^c^** |
| **Model I^d^** | 0.070 | 0.02, 0.12 | 0.01 | **-** | **-** | **-** | **-** | **-** | **-** |
| **Model II^d^** | **-** | **-** | **-** | -0.060 | -0.13, 0.01 | 0.08 | **-** | **-** | **-** |
| **Model III^d^** | **-** | **-** | **-** | **-** | **-** | **-** | 0.011 | -0.05, 0.07 | 0.73 |
| **Model IV^e^** | 0.074 | 0.02, 0.13 | 0.007 | -0.062 | -0.13, 0.01 | 0.07 | **-** | **-** | **-** |
| **Model V^f^** | 0.114 | 0.04, 0.19 | 0.002 | **-** | **-** | **-** | -0.073 | -0.15, 0.01 | 0.07 |
| **Models** | **Air quality improvement in** **NO_2_^a^** | | | **Recent NO_2_ exposure^a^** | | | **Remote NO_2_ exposure^a^** | | |
|  | **β^b^** | **95% CI** | **p^c^** | **β^b^** | **95% CI** | **p^c^** | **β^b^** | **95% CI** | **p^c^** |
| **Model I^d^** | 0.060 | 0.005, 0.12 | 0.03 | **-** | **-** | **-** | **-** | **-** | **-** |
| **Model II^d^** | **-** | **-** | **-** | -0.041 | -0.10, 0.02 | 0.20 | **-** | **-** | **-** |
| **Model III^d^** | **-** | **-** | **-** | **-** | **-** | **-** | 0.004 | -0.06, 0.07 | 0.91 |
| **Model IV^e^** | 0.088 | 0.03, 0.15 | 0.004 | -0.077 | -0.15, -0.01 | 0.03 | **-** | **-** | **-** |
| **Model V^f^** | 0.137 | 0.05, 0.22 | 0.002 | **-** | **-** | **-** | -0.116 | -0.22, -0.01 | 0.03 |

Abbreviations: WHIMS-ECHO, Women’s Health Initiative Memory Study-Epidemiology of Cognitive Health Outcomes; TICSm, modified Telephone Interview for Cognitive Status; CVLT, California Verbal Learning Tests; PM_2.5_, fine particulate matter; NO_2_, nitrogen dioxide

^a^ Recent exposures were the 3-year average exposures estimated at the WHIMS-ECHO enrollment. Remote exposures were the 3-year average exposures estimated 10 years before the WHIMS-ECHO enrollment. Air quality improvement was defined as reduction from the remote to recent exposures over the 10-year period.

TICSm analyses: IQR_PM2.5_ _improvement_ = 1.79 µg/m^3^; IQR_NO2_ _improvement_ = 3.92 ppb; IQR _recent PM2.5_ = 2.88 µg/m^3^; IQR _recent NO2_ = 6.19 ppb; IQR _remote PM2.5_ = 3.24 µg/m^3^; IQR _recent NO2_ = 9.42 ppb;

CVLT analyses: IQR_PM2.5_ _improvement_ = 1.79 µg/m^3^; IQR_NO2_ _improvement_ = 3.97 ppb; IQR _recent PM2.5_ = 2.79 µg/m^3^; IQR _recent NO2_ = 6.28 ppb; IQR _remote PM2.5_ = 3.26 µg/m^3^; IQR _recent NO2_ = 9.47 ppb.

^b^ β (95% CI) = regression coefficient (95% confidence interval) estimating the increase in TICSm score or CVLT score per year for each interquartile range (IQR) increase of air quality measures, adjusting for spatial random effect, WHIMS-ECHO enrollment year, age, follow-up year, age interaction with follow-up year, time-varying propensity scores, demographic variables (geographic region, and race/ethnicity), socioeconomic factors (education, income, employment status) and neighborhood socioeconomic characteristics, lifestyle factors (smoking, drinking and physical activities), prior hormone use, hormone therapy assignment, cardiovascular risk factors (hypertension, diabetes and hypercholesterolemia), depression, body mass index, and cardiovascular disease histories. Positive coefficients represent slower decline associated with greater air quality improvement or air quality measures.

^c^ P values were calculated using Wald t-tests.

^d^ Model I included air quality improvement measure only. Model II included recent air pollution exposure only. Model III included remote air pollution exposure only.

^e^ Model IV included both air quality improvement and recent exposure in the same model.

^f^ Model V included both air quality improvement and remote exposure in the same model.
