# Supplementary material for: Air quality improvement and cognitive decline in community-dwelling older women in the United States: A longitudinal cohort study
Source: PLoS Med. 2022 Feb 3;19(2):e1003893. doi: 10.1371/journal.pmed.1003893 (PMC8812844; doi:10.1371/journal.pmed.1003893)
Supplement: S9 Table — AQ, air quality. (DOCX) [file pmed.1003893.s020.docx]

**S9 Table. Summary of the Associations between Air Quality Improvement and Quadratic Term of Cognitive Decline.**

|  |  | **Air quality improvement in PM_2.5_^a^** | | **Air quality improvement in NO_2_^a^** | |
| --- | --- | --- | --- | --- | --- |
| **Cognitive outcomes** | **N** | **β^b,c^** | **p^d^** | **β^b,c^** | **p^d^** |
| **TICSm** | 2232 | -0.001 | 0.81 | -0.0002 | 0.97 |
| **CVLT** | 1721 | 0.017 | 0.11 | 0.014 | 0.23 |

Abbreviations: WHIMS-ECHO, Women’s Health Initiative Memory Study-Epidemiology of Cognitive Health Outcomes; TICSm, modified Telephone Interview for Cognitive Status; CVLT, California Verbal Learning Tests; PM_2.5_, fine particulate matter; NO_2_, nitrogen dioxide

^a^ Recent exposures were the 3-year average exposures estimated at the WHIMS-ECHO enrollment. Remote exposures were the 3-year average exposures estimated 10 years before the WHIMS-ECHO enrollment. AQ improvement was defined as reduction from the remote to recent exposures over the 10-year period.

^b^ β represents AQ improvement association with quadratic term of follow-up year, per each IQR increase of AQ improvement (IQR_PM2.5_ = 1.79 µg/m^3^ for both TICSm and CVLT analytic samples; IQR_NO2_ = 3.92 ppb for TICSm analytic sample and 3.97 ppb for CVLT analytic sample).

^c^ Models adjusted for spatial random effect, WHIMS-ECHO enrollment year, age, follow-up year, age interaction with follow-up year, AQ improvement interaction with follow-up year, quadratic term of follow-up year, demographic variables (geographic region and race/ethnicity), socioeconomic factors (education, income, employment status) and neighborhood socioeconomic characteristics, lifestyle factors (smoking, drinking and physical activities), prior hormone use, hormone therapy assignment, cardiovascular risk factors (hypertension, diabetes and hypercholesterolemia), depression, body mass index, cardiovascular disease histories, and time-varying propensity scores.

^d^ P values were calculated using Wald t-tests.
