# Supplementary material for: Air quality improvement and cognitive decline in community-dwelling older women in the United States: A longitudinal cohort study
Source: PLoS Med. 2022 Feb 3;19(2):e1003893. doi: 10.1371/journal.pmed.1003893 (PMC8812844; doi:10.1371/journal.pmed.1003893)
Supplement: S10 Table — AQ, air quality. (DOCX) [file pmed.1003893.s021.docx]

**S10 Table. Summary of the Associations between Air Quality Improvement and Cognitive Declines, with the Non-Linear Change in Cognitive Trajectories**

| **A) Associations with declines in general cognitive ability (N=2232)** | | | | | | |
| --- | --- | --- | --- | --- | --- | --- |
|  | **Air quality improvement in PM_2.5_^a^** | | | **Air quality improvement in NO_2_^a^** | | |
| **Models** | **β^b^** | **95% CI** | **p^c^** | **β^b^** | **95% CI** | **p^c^** |
| **Model I^d^** | 0.026 | 0.001, 0.05 | 0.04 | 0.034 | 0.01, 0.06 | 0.005 |
| **Model II^e^** | 0.024 | 0.0001, 0.05 | 0.05 | 0.033 | 0.01, 0.06 | 0.006 |
| **B) Associations with declines in episodic memory (N=1721)** | | | | | | |
|  | **Air quality improvement in PM_2.5_^a^** | | | **Air quality improvement in NO_2_^a^** | | |
| **Models** | **β^b^** | **95% CI** | **p^c^** | **β^b^** | **95% CI** | **p^c^** |
| **Model I^d^** | 0.070 | 0.02, 0.12 | 0.01 | 0.060 | 0.005, 0.12 | 0.03 |
| **Model II^e^** | 0.061 | 0.01, 0.12 | 0.03 | 0.051 | -0.004, 0.11 | 0.07 |

Abbreviations: WHIMS-ECHO, Women’s Health Initiative Memory Study-Epidemiology of Cognitive Health Outcomes; TICSm, modified Telephone Interview for Cognitive Status; CVLT, California Verbal Learning Tests; PM_2.5_, fine particulate matter; NO_2_, nitrogen dioxide

^a^ Recent exposures were the 3-year average exposures estimated at the WHIMS-ECHO enrollment. Remote exposures were the 3-year average exposures estimated 10 years before the WHIMS-ECHO enrollment. Air quality improvement was defined as reduction from the remote to recent exposures over the 10-year period.

^b^ β (95% CI) = regression coefficient (95% confidence interval) estimating the increase in TICSm score or CVLT score per year for each interquartile range (IQR) increase of air quality improvement (IQR_PM2.5_ = 1.79 µg/m^3^ for both analytic samples; IQR_NO2_ = 3.92 ppb for TICSm analytic sample and 3.97 ppb for CVLT analytic sample). Positive coefficients represent slower decline associated with greater air quality improvement.

^c^ P values were calculated using Wald t-tests.

^d^ Model I: adjusted for spatial random effect, WHIMS-ECHO enrollment year, age, follow-up year, age interaction with follow-up year, demographic variables (geographic region and race/ethnicity), socioeconomic factors (education, income, employment status) and neighborhood socioeconomic characteristics, lifestyle factors (smoking, drinking and physical activities), prior hormone use, hormone therapy assignment, cardiovascular risk factors (hypertension, diabetes and hypercholesterolemia), depression, body mass index, cardiovascular disease histories, and time-varying propensity scores.

^e^ Model II: adjusted for those in Model I, as well as quadratic term of follow-up year.
