# Supplementary material for: Air quality improvement and cognitive decline in community-dwelling older women in the United States: A longitudinal cohort study
Source: PLoS Med. 2022 Feb 3;19(2):e1003893. doi: 10.1371/journal.pmed.1003893 (PMC8812844; doi:10.1371/journal.pmed.1003893)
Supplement: S11 Table — AQ, air quality. (DOCX) [file pmed.1003893.s022.docx]

**S11 Table. Summary of the Associations with Cognitive Trajectory Slopes, Evaluating Non-Linear Associations with Air Quality Improvement**

| **A) Associations between linear and quadratic terms of PM_2.5_ improvement^a^ with slope of cognitive trajectory** | | | | | | | |
| --- | --- | --- | --- | --- | --- | --- | --- |
|  |  | **Air quality improvement** | | | **Air quality improvement^2^** | | |
| **Cognitive outcomes** | **N** | **β_1_^b^** | **95% CI** | **p^d^** | **β_2_^c^** | **95% CI** | **p^d^** |
| **TICSm** | 2232 | 0.064 | 0.02, 0.11 | 0.004 | -0.012 | -0.02, -0.0005 | 0.04 |
| **CVLT** | 1721 | 0.081 | -0.02, 0.18 | 0.11 | -0.003 | -0.03, 0.02 | 0.83 |
| **B) Associations between linear and quadratic terms of NO_2_ improvement^a^ with slope of cognitive trajectory** | | | | | | | |
|  |  | **Air quality improvement** | | | **Air quality improvement^2^** | | |
| **Cognitive outcomes** | **N** | **β_1_^b^** | **95% CI** | **p^d^** | **β_2_^c^** | **95% CI** | **p^d^** |
| **TICSm** | 2232 | 0.069 | 0.02, 0.12 | 0.004 | -0.010 | -0.02, 0.002 | 0.09 |
| **CVLT** | 1721 | 0.057 | -0.05, 0.17 | 0.31 | 0.001 | -0.02, 0.03 | 0.94 |

Abbreviations: WHIMS-ECHO, Women’s Health Initiative Memory Study-Epidemiology of Cognitive Health Outcomes; TICSm, modified Telephone Interview for Cognitive Status; CVLT, California Verbal Learning Tests; PM_2.5_, fine particulate matter; NO_2_, nitrogen dioxide; CI, confidence interval

^a^ Recent exposures were the 3-year average exposures estimated at the WHIMS-ECHO enrollment. Remote exposures were the 3-year average exposures estimated 10 years before the WHIMS-ECHO enrollment. Air quality (AQ) improvement was defined as reduction from the remote to recent exposures over the 10-year period.

^b^ β_1_ represents AQ improvement association with slope of cognitive trajectory, per each interquartile range (IQR) increase of AQ improvement (IQR_PM2.5_ = 1.79 µg/m^3^ for both TICSm and CVLT analytic samples; IQR_NO2_ = 3.92 ppb for TICSm analytic sample and 3.97 ppb for CVLT analytic sample). Positive coefficients represent slower decline associated with greater AQ improvement.

^c^ β_2_ represents AQ improvement^2^ association with slope of cognitive trajectory, per the square of each IQR increase of AQ improvement. Positive coefficients represent a convex relationship between AQ improvement and cognitive trajectory slope.

^d^ P values were calculated using Wald t-tests, with models included both linear and quadratic terms of AQ improvement and adjusted for spatial random effect, WHIMS-ECHO enrollment year, age, follow-up year, age interaction with follow-up year, demographic variables (geographic region and race/ethnicity), socioeconomic factors (education, income, employment status) and neighborhood socioeconomic characteristics, lifestyle factors (smoking, drinking and physical activities), prior hormone use, hormone therapy assignment, cardiovascular risk factors (hypertension, diabetes and hypercholesterolemia), depression, body mass index, cardiovascular disease histories, and time-varying propensity scores.
