# Supplementary material for: Air quality improvement and cognitive decline in community-dwelling older women in the United States: A longitudinal cohort study
Source: PLoS Med. 2022 Feb 3;19(2):e1003893. doi: 10.1371/journal.pmed.1003893 (PMC8812844; doi:10.1371/journal.pmed.1003893)
Supplement: Attachment — Submitted filename: Response to Reviewers.docx [file pmed.1003893.s023.docx]

**Comments from the Academic Editor:**

**This is a very good attempt at trying to relate improvement in environmental pollution with cognitive decline. They have tried to control demographic, lifestyle and clinical factors. It is very likely that not all confounding factors can be accounted for in such a study. The question that comes up is, do the people living in an environment that is improving differ systematically from those who live in an environment that is not?**

- The air quality (AQ) improvement measures used in our study were defined as reduction from remote to recent exposures over the 10-year period. They were treated as continuous variables in our study.
- We compared the distributions of AQ improvement by population characteristics and presented the results in Table 2. Overall, non-Hispanic White women experienced less AQ improvement, while women who were older than 80, reported higher income, or resided in the Northeast and West experienced greater reductions in ambient PM_2.5_ and NO_2_, as compared to their counterparts (Table 2).

**One can account for edu and income, but is that enough? Do they have a generally healthy lifestyle? Do they exercise more? Do they eat healthily? Some of these are difficult to control for?**

- AQ improvement did not differ across smoking or physical activity status; however, non-drinkers experienced less reduction in ambient NO_2_, compared to current or past drinkers (Table 2). We did not compare the distribution of AQ improvement by dietary habit.
- We adjusted for socio-demographics (age; geographic region; race/ethnicity; education; income; employment status; neighborhood socioeconomic characteristics), lifestyle factors (smoking; alcohol; physical activity), or clinical characteristics (BMI; depressive symptoms; diabetes; hypercholesterolemia; hypertension; CVD; hormone therapy) in our fully-adjusted models to control for potential confounding.

**The other issue is that the improvement in the environment occurred over the 10 years prior to the recruitment. Has that improvement continued through the study? Did other areas catch up?**

- We focused on using AQ improvement over the 10 years prior to the recruitment because we wanted to avoid the methodological concerns on the temporality between the defined period of AQ improvement and the concurrently observed slower cognitive decline during the follow-up.
- We have added the following to the Methods (estimation of air pollution exposure sub-section) to make this clearer: *“We focused on AQ improvement over the 10 years prior to recruitment in order to avoid methodological concerns on the temporality between the defined period of AQ improvement and the concurrently observed slower cognitive decline during the follow-up.”*

**Requests from the editors:**

**General comments:**

**Throughout the paper, please adapt reference call-outs to the following style: "... Alzheimer’s disease (AD) neuropathologies [17,18].” (noting the absence of spaces within the square brackets).**

- We have updated the reference call-outs.

**Please avoid using ‘average’ - please specify.**

- We have avoided using “average” in describing the associations estimated from models throughout the manuscript as follows:
  - “*Linear mixed effect models were used to examine the associations between improved AQ and the ~~average~~ rates of cognitive declines in TICSm and CVLT trajectories…*” (Abstract)
  - “*We used linear mixed effect models to examine whether AQ improvement before WHIMS-ECHO enrollment was associated with ~~average~~ decline rates in the TICSm and CVLT trajectories during the follow-up.*” (Methods, Statistical analyses section)
  - “*β (95% CI) = regression coefficient (95% confidence interval) estimating the ~~average~~ increase in TICSm score or CVLT score per year for each interquartile range (IQR) of increase of air quality improvement …*” (Footnotes of Table 3 and S5-S8 Tables)

**Please replace "subject" with participant, patient, individual, or person.**

- We have replaced “subject” throughout.

**Thank you for providing a data availability statement. PLOS Medicine requires that the de-identified data underlying the specific results in a published article be made available, without restrictions on access, in a public repository or as Supporting Information at the time of article publication, provided it is legal and ethical to do so. If the data will be freely available upon request, please state the owner of the data set and specific contact information for data requests (web or email address). Please note that a study author cannot be the contact person for the data.**

- According to PLOS Medicine guideline, we have revised the data statement as follows: “*Data Statement: ~~Access to all data elements used in this study may be made available on the WHI website (www.whi.org) following the established WHI policies.~~* *Data, codebook, and analytic code used in this report may be accessed in a collaborative mode as described on the Women’s Health Initiative website (https://urldefense.com/v3/__http://www.whi.org__;!!LIr3w8kk_Xxm!76lJ1eegY7-QWP88j12lZ9GmitZamnptj8mzyvGVq9XEfIBi-3CzQc8cO5RNwf4$ ).*”

**Title: Please revise your title according to PLOS Medicine's style. Your title must be nondeclarative. Please place the study design in the subtitle (ie, after a colon). We suggest “Association between air quality improvement and changes in cognitive function in community-dwelling older women: A longitudinal cohort study“**

- We have changed the title to “Association between air quality improvement and slower cognitive decline in community-dwelling older women: A longitudinal cohort study”

**Abstract:**

**Abstract Background: Please revise to “Air quality (AQ) has been associated with…”, and please temper assertions of primacy by adding “to the best of our knowledge” or similar. The final sentence should clearly state the study question.**

- We have made the suggested changes.

**Abstract Methods and Findings:**

**Please provide brief demographic details of the study population (e.g. sex, age, ethnicity, etc)**

- We had already included information on sex and age in the initial submission. In the revision, we have specified that this was a cohort of predominantly non-Hispanic White women.

**Please clarify what is represented by the units used for the numbers presented in the abstract e.g. (βPM2.5=0.026/year per IQR=1.79 µg/m3). It may be easier to describe this.**

- We have elaborated that these represent the rate of slower cognitive decline per year associated with each increment of air quality improvement. For example: *“(β_PM2.5 improvement_=0.026 per ~~/~~year ~~per~~for improved PM_2.5_ by each ~~increment in~~ IQR=1.79 µg/m^3^ reduction)”*

**Please revise the sentence beginning “ Individual-level improved AQ calculated…”**

- We have revised this as follows: *“Individual-level improved AQ was calculated as the reduction from remote to recent exposures.”*

**Line 57 - Please make clear that the estimates were 3-year averages**

- We have revised this sentence as follows: *“Estimates were ~~averaged over~~ aggregated to the 3-year ~~periods~~ average immediately preceding (recent exposure) and 10 years prior to (remote exposure) WHIMS-ECHO enrollment.”*

**Please specify the important dependent variables that are adjusted for in the analyses.**

- We have expanded the covariate list as follows: *“Linear mixed effect models were used to examine the associations between improved AQ and the average rates of cognitive declines in TICSm and CVLT trajectories, adjusting for sociodemographic (age; geographic region; race/ethnicity; education; income; employment), lifestyle (prior hormone use; hormone therapy assignment; depression; physical activity; smoking; alcohol), and clinical characteristics (cardiovascular disease; hypercholesterolemia; hypertension; diabetes; body mass index).”*

**Please clarify whether the results presented are adjusted analyses, and please specify the comparison group.**

- We have specified that the reported results were from models adjusting for covariates.
- All associations were reported per one interquartile range increase of the AQ improvement measure. Therefore, there is no comparison group in our analyses and we did not provide this information.
- We have attempted to make this clearer by reporting the associations as “greater improvement in AQ was associated with slower cognitive decline” rather than “AQ improvement was associated with slower cognitive decline” throughout the manuscript.

**Please define ppb at first use**

- We have defined ppb at first use.

**In the last sentence of the Abstract Methods and Findings section, please describe 2-3 of  the main limitation(s) of the study's methodology."**

- We have added the following sentence: *“The main limitations of this study include use of measurement error in exposure estimates, potential unmeasured confounding, and limited generalizability.”*

**Abstract Conclusions:**

**Please begin your Abstract Conclusions with "In this study, we observed ..." or similar, to summarize the main findings from your study, and expand a little on the implications of your study without overstating your conclusions.**

- We have changed this sentence to: *“In this study, we found that greater improvement in l~~L~~ong-term ~~improvement in~~ AQ in late-life was associated with slower cognitive declines in older women. This novel observation strengthens the epidemiologic evidence of an association between air pollution and cognitive aging.”*

**Author Summary:**

**At this stage, we ask that you include a short, non-technical Author Summary of your research to make findings accessible to a wide audience that includes both scientists and non-scientists. The Author Summary should immediately follow the Abstract in your revised manuscript. This text is subject to editorial change and should be distinct from the scientific abstract. Please see our author guidelines for more information:** [**https://urldefense.com/v3/__https://journals.plos.org/plosmedicine/s/revising-your-manuscript*loc-author-summary__;Iw!!LIr3w8kk_Xxm!4uNvMN_eLXDfLkzyekgMV0GL1AYI4u78yLHWhIlDqN-xqy8bfxIVheW7U3LYJQc$**](https://urldefense.com/v3/__https:/journals.plos.org/plosmedicine/s/revising-your-manuscript*loc-author-summary__;Iw!!LIr3w8kk_Xxm!4uNvMN_eLXDfLkzyekgMV0GL1AYI4u78yLHWhIlDqN-xqy8bfxIVheW7U3LYJQc$)

**In the final bullet point of ‘What Do These Findings Mean?’, please describe the main limitations of the study in non-technical  language.**

- We have included the author summary in the revised manuscript.

**Introduction:**

**Please indicate whether your study is novel and how you determined that (being careful to temper assertions of primacy by adding “to the best of our knowledge” or similar). If there has been a systematic review of the evidence related to your study, please refer to and reference that review and indicate whether it supports the need for your study.**

- In the first paragraph of the Introduction, we had cited a systematic review of the evidence on air pollution exposure and cognitive decline, stating that the findings have been mixed.
- However, no studies had previously looked at air quality improvement and slower cognitive decline. Therefore, we have added the following sentence to the Introduction (3^rd^ paragraph): *“To the best of our knowledge, no previous studies have explored whether AQ improvement may be associated with cognitive function.”*

**Methods:**

**Did your study have a prospective protocol or analysis plan? Please state this (either way) early in the Methods section. If a prospective analysis plan (from your funding proposal, IRB or other ethics committee submission, study protocol, or other planning document written before analyzing the data) was used in designing the study, please include the relevant prospectively written document with your revised manuscript as a Supporting Information file to be published alongside your study, and cite it in the Methods section. A legend for this file should be included at the end of your manuscript. If no such document exists, please make sure that the Methods section transparently describes when analyses were planned, and if/when reported analyses differed from those that were planned. Changes in the analysis-- including those made in response to peer review comments-- should be identified as such in the Methods section of the paper, with rationale. If a reported analysis was**

**performed based on an interesting but unanticipated pattern in the data, please be clear that the analysis was data-driven.**

- We have added the following sentence to the Methods (Study design and population subsection): *“Our study did not employ a prospective protocol. Analyses were first planned and performed in April 2020, and before the submission the completed manuscript was revised by two anonymous reviewers assigned by the WHI Publications and Presentations Committee.”*

**Thank you for providing your STROBE guideline. Please add the following statement, or similar, to the Methods: "This study is reported as per the Strengthening the Reporting of Observational Studies in Epidemiology (STROBE) guideline (S1 Checklist)." When completing the checklist, please use section and paragraph numbers, rather than page numbers which will likely no longer correspond to the appropriate sections after copy-editing.**

- We have added the suggested statement to the Methods section (1^st^ paragraph)
- We have also updated the STROBE checklist using section and paragraph numbers.

**Results:**

**Please incorporate S1 Table into your Results section as Table 1, showing the baseline characteristics of the study population.**

- We put the S1 Table as the new Table 1 and renamed the other Tables accordingly.

**Line 214 - please clarify what is represented by ‘±2.7’ (presumably SD)**

- We have specified this as the SD in the sentence.

**Line 230 - please be careful to avoid causative language; please revise sentence beginning “Older women residing…” to “Residing in locations with greater AQ improvement was associated with slower rates of decline... “ or similar**

- We have made the suggested changes.

**Line 232 - please revise sentence beginning “For each IQR increment of improved AQ…” to “Each IQR increment … was associated with…“ or similar**

- We have made the suggested changes.

**Discussion:**

**Line 347 - please temper assertions of primacy by adding “to the best of our knowledge” or similar. Please also revise the use of ‘causal’ on lines 347 and 362; we suggest ‘strengthen the evidence' or similar.**

- We have made the suggested changes.

**Figures:**

**Please consider avoiding the use of red and green together in order to make your figure more accessible to those with colour blindness (e.g. Fig 2).**

- We have changed the colors to be black, blue and red.

**Please indicate in the figure caption the meaning of the bars and whiskers in Figure 3.**

- We have made the suggested changes.

**Please define all abbreviations used in the figure legend of each figure.**

- We have made the suggested changes.

**Tables:**

**Please define all abbreviations used in the table legend of each table.**

- We have made the suggested changes.

**When a p value is given, please specify the statistical test used to determine it in the table legend.**

- We have made the suggested changes.

**Table 2, S3 Table - Please also provide the unadjusted analyses/models.**

- In response to this comment and others raised by Reviewer #2, we have slightly restructured our models to incorporate unadjusted analyses. In our original submission, we had our base model (original Model I) which included the essential set of covariates that needed to be adjusted for in our models to examine the average decline rates in the TICSm and CVLT trajectories during the follow-up and to account for spatiotemporal confounding and selective attrition over the study period. We then had our fully adjusted model (original Model II) which was our Model I plus all of the confounders (demographic variables; socioeconomic factors; lifestyle factors; clinical characteristics).
- In the revision, we kept our Model I the same, but we have added a new model to Table 2 (now renamed as Table 3), which is referred to as the new Model II. This model included the covariates in Model I plus any variables that researchers in the field would agree to be predictors of both air pollution exposure and brain aging (e.g., geographic region; race/ethnicity; socioeconomic factors [education, income, employment status] and neighborhood socioeconomic characteristics; and lifestyle factors [smoking, drinking, physical activities]). Additionally, our original Model II has now been renamed to Model III. This Model III included the covariates in Model II plus any variables that some researchers may argue could theoretically be mediators of the pathway between air pollution exposure and brain aging (e.g. prior hormone use; hormone therapy assignment; depression; cardiovascular risk factors [hypertension, diabetes and hypercholesterolemia]; body mass index; cardiovascular disease histories). Our results indicate that the estimated associations did not vary much across models.
- The original S3 Table (now renamed as S5 Table) summarized the sensitivity analyses results with adjustment of covariates assessed at the WHIMS-ECHO enrollment or changes from WHI Inception to WHIMS-ECHO enrollment, rather than the covariates assessed at WHI inception which were presented in Table 3 (originally Table 2). Results of both models presented in S5 table (originally S3 table) would be compared to the fully adjusted model results (Model III) in the new Table 3. Unadjusted models are not presented, because the results were not relevant to such comparison.

**S5 Table - please clarify how the models differ in the table legend.**

- The results presented in S5 Table (now renamed as S7 Table) were to explore whether our findings could be explained by regression to the mean in air quality measures, as described in the *Statistical Analyses* section (2^nd^ paragraph, third sensitivity analysis).
- We edited the table legend of S5 Table (now renamed as S7 Table) as bellows to clarify differences among these models: “*^d^ Model I included air quality improvement measure only. Model II included recent air pollution exposure only. Model III included remote air pollution exposure only.*

*^e^ Model IV i~~I~~ncluded both air quality improvement and recent exposure in the same model.*

*^f^ Model V i~~I~~ncluded both air quality improvement and remote exposure in the same model.*”

**References:**

**Please ensure that journal name abbreviations match those found in the National Center for Biotechnology Information (NCBI) databases, and are appropriately formatted and capitalised.**

**Please also see** [**https://urldefense.com/v3/__https://journals.plos.org/plosmedicine/s/submission-guidelines*loc-references__;Iw!!LIr3w8kk_Xxm!4uNvMN_eLXDfLkzyekgMV0GL1AYI4u78yLHWhIlDqN-xqy8bfxIVheW74deINks$**](https://urldefense.com/v3/__https:/journals.plos.org/plosmedicine/s/submission-guidelines*loc-references__;Iw!!LIr3w8kk_Xxm!4uNvMN_eLXDfLkzyekgMV0GL1AYI4u78yLHWhIlDqN-xqy8bfxIVheW74deINks$)  **for further details on reference formatting.**

- We have gone through and updated some of the references.

**Comments from the reviewers:**

**Reviewer #1: This study attempts to leverage a nationwide cohort of community dwelling older women with individual-level air pollution exposure estimates (1996-2012) and annual assessments of late-life cognitive function (2008−2018). The authors hypothesise that improved AQ, as indicated by reductions in PM2.5 and NO2, is associated with slower rate of cognitive decline in older women.**

**Comments:**

**The STROBE checklist has been suitably provided in the supplementary material.**

**"We conducted a longitudinal study on a geographically diverse cohort of community dwelling older women (N=2880; aged 74-92) enrolled in the Women's Health Initiative (WHI) Memory Study (WHIMS)-Epidemiology of Cognitive Health Outcomes (WHIMS-ECHO) study ... This resulted in a final analytic sample of 2232 women for the analyses on general cognitive status, a subset of which (n=1721) was used for the analyses on episodic memory (Fig 1A)."**

**Can the authors please comment on whether they believe this cohort to be representative of the wider population? I.E. Are there any potential causes of bias that should be highlighted?**

- In the manuscript, we compared the women excluded due to missing AQ measures or relevant covariates (Fig 1A) with women included in our analyses. We found that these two groups did differ by age, socioeconomic status, region of residence, race/ethnicity, and alcohol use (Table 1). We adjusted for these covariates in our models. However, we cannot completely rule out the possibility of selection bias. Therefore, in the revised manuscript, we added an additional limitation to address this: *“Sixth, because our study sample came from a non-random selective process (Figure 1A), we could not completely rule out the possibility of selection biases.”*
- With regards to external validity and whether this sample is representative of the general population, we had included a limitation that our findings were not generalizable to men or younger women.

**"WHIMS-ECHO participants received annual neuropsychological assessments via centralized telephone-administered cognitive interviews conducted by trained and certified staff."**

**Can the authors please discuss the potential for self-reporting bias in the study limitations?**

- Self-reporting bias is a concern when use of an objective measure (e.g., psychological tests) of the variable of interest is not available and the researcher must rely on use of a subjective measure (e.g., survey; questionnaire; interview) as a proxy instead [1]. In our study, cognitive function was assessed based on performance on the objective TICSm and CVLT cognitive tests which were conducted over the telephone. Self-reporting bias would not be a concern in our study because we used an objective measure to gather information on their cognitive function and we did not rely on the participant to self-report their cognitive function.
- These telephone-based cognitive assessments have been shown to be both reliable and valid [2].

**It is noted that, with respect to covariates:  "Good reliability and validity of both the self-reported medical histories and the physical measures have been documented (37-39)."**

**"Participants were asked 16 questions with items assessing several cognitive functions.  The TICSm score (0-50) was defined as the total number of correct responses, with higher scores indicating better cognitive functioning."**

**Can these questions and scoring computation please be provided in the supplementary material?**

- Unfortunately, we cannot reproduce the TICSm in the appendix because it is copyrighted.
- Additionally, after reviewing our description of the TICSm we realized our description may have been confused, as the TICSm is a test rather than a survey. Therefore, we have updated the description in the revised manuscript as follows: *“~~Participants were asked 16 questions with items assessing several cognitive functions.~~ The TICSm includes 16 cognitive test items from different cognitive function domains, including recall of a list of words, generating a list of nouns as quickly as possible, naming common items, and counting backwards. The TICSm score (0-50) was defined as the total number of correct responses,* *with test items with multiple parts contributing a corresponding number of points* *~~with~~ and higher scores indicating better cognitive functioning.”* (Methods, 2^nd^ paragraph)

**"In the present study, we used all longitudinal data collected from telephone based assessments until June 2018 (29)."**

**Can this be extended to 2020?**

- Although the WHIMS-ECHO participants are still being followed up until now, we do not have access to the cognitive assessment data collected after June 2018 at this time.

**"We used regionalized universal kriging models to estimate annual concentrations (1996-2012) of fine particulate matter (PM2.5) and nitrogen dioxide (NO2) at residential locations. Estimates were averaged over 3-year periods immediately preceding (recent exposure) and 10 years prior to (remote exposure) WHIMS ECHO enrollment."**

**Did the authors consider including a more current AQ assessment, i.e. after enrolment, as well as these two time points?**

- No, we did not consider this. Had we included a more current AQ assessment covering years after enrollment, the resulting study design would raise a methodological concern with the temporality between the defined period of AQ improvement and the concurrently observed slower cognitive decline during the follow-up.
- Lack of temporality was one limitation noted in previous studies [3,4] on the health benefits of AQ improvement. In our study, we defined AQ improvement based on the difference in the estimated exposures between the WHIMS-ECHO enrollment and remote period, which greatly reduced this methodological uncertainty and ensures the temporality between AQ improvement and slower cognitive decline.

**"We used linear mixed effect models to examine whether AQ improvement before WHIMS-ECHO enrollment was associated with average decline rates in the TICSm and CVLT trajectories during the follow-up".**

**This is a technically appropriate methodology, assuming linearity holds.**

- In our prior hypothesis, we did not make assumptions regarding linearity of effects. However, we employed a linear modeling approach to increase statistical power in view of our limited a priori knowledge of which specific models would capture possible departures from linearity.
- It is possible that there are factors that would cause non-linear changes in cognitive decline, e.g., non-linear aging processes, selective attrition, advanced neuropathologies, or heterogeneity in cognitive decline trajectories. However, we were not able to detect any such non-linear effects in sensitivity analyses.
- For example, we included a quadratic term of follow-up year (denoted as year^2^) in the models and evaluated if AQ improvement was associated with the quadratic change of cognitive trajectory. We did not find significant associations between improvement in either PM_2.5_ or NO_2_ with quadratic change of TICSm or CVLT trajectories (all p-values > 0.10 for the interaction term AQ improvement*year^2^).
- We further examined if the associations between AQ improvement and the linear term of follow-up year remained with year^2^ added in models. The associations with the TICSm trajectory slope were similar, while associations with the CVLT trajectory slope were slightly attenuated for both PM_2.5_ and NO_2_ improvement. The results are in a new supplementary table (S10 Table).
- We also evaluated if there are non-linear AQ improvement effects on the slope of cognitive decline. We used a quadratic term of AQ improvement (denoted as AQ improvement^2^) in the model and tested if the interaction term AQ improvement^2^*year was significant. Except for some evidence showing the estimate of PM_2.5_ improvement^2^ was statistically significant on TICSm trajectory slope (p = 0.04 for PM_2.5_ improvement^2^*year), the overall results did not support the non-linear effects of AQ improvement on cognitive trajectory slopes. These results are summarized in a new supplementary table (S11 Table).
- We added these sensitivity analyses to Methods and Results as follows:
  - “*Sixth, we conducted additional analyses with interactions between the measures of AQ improvement and a quadratic term of follow-up year in the adjusted models to examine whether AQ improvement was associated with non-linear changes in cognitive function. We then evaluated whether the associations between AQ improvement and linear trajectory slopes were sensitive to incorporating the non-linear changes of cognitive function in the analyses with a quadratic term of follow-up year. Finally, we assessed the non-linear AQ improvement effect on cognitive trajectory slope by examining the interaction between a quadratic term of AQ improvement and follow-up year.*” (Methods, Statistical analyses section, 2^nd^ paragraph)
  - “*We did not find significant associations between AQ improvement and quadratic change in cognitive function (all p-values > 0.10,* *S9 Table). With the quadratic term of follow-up year included in models, the associations between AQ improvement and TICSm trajectory slope were similar to the estimates in the main analyses, while the estimated associations with CVLT trajectory slopes were slightly attenuated for both PM_2.5_ and NO_2_ improvement (S10 Table). Except for some evidence supporting the quadratic term for PM_2.5_ improvement on TICSm trajectory slope (p=0.04 for the interaction between the quadratic term of PM_2.5_ improvement and follow-up year, S11 Table), the overall results did not suggest non-linear effects of AQ improvement on cognitive trajectory slopes (S11 Table).*” (Results, 2^nd^ to last paragraph)

**Overall, this is a clearly written article, with excellent communication of information via figures.**

**The authors have included an extensive array of potential confounders within the model, and completed a thorough set of sensitivity analyses demonstrating the robustness of the study outcomes.**

**Furthermore, the study limitations have been acknowledged suitably within the discussion section of the manuscript.**

**Reviewer #2: The study of Younan et al examines whether air pollution improvement of NO2 and PM2.5 over a 10-years period, is associated with cognitive improvement in elderly women from US. They used data from 2,000 women enrolled in the Women's Health Initiative Memory Study-Epidemiology of Cognitive Health Outcomes who provided on general cognitive status and episodic memory decline. Authors observed that greater air quality improvement for NO2 and PM2.5 was associated with slower decline in both outcomes. The study is well-designed and well-written. Main strengths include the repeated outcomes measurements and the large sample size. Hereby I include some comments:**

**Major comments:**

- **Indoor air quality is not considered in the present study and this may have a great impact in the association since elderly women spent most of the time indoors.**
- The reviewer is correct that elderly women likely spend most of their time indoors; however, there are a few points of clarification we would like to raise. First, the vast majority of air pollution epidemiological studies have focused on individual exposure to air pollutants from the outdoor sources because these levels are much more important from the public health perspective since they can be regulated by the Environmental Protection Agency, and indoor sources cannot. Second, although older people may stay inside more and may not be outdoors as often, ambient concentrations contribute to indoor exposures, as well, due to the infiltration of ambient pollution into indoor environments [5]. Lastly, it is important to note that several studies have consistently shown that sources of indoor air pollution (e.g., environmental tobacco smoke; fireplaces; wood/gas stoves) do not confound the exposure effect of outdoor air pollution [6,7].
- **Authors are aware that they are not considering other features of the urban environment such as green spaces nor noise. It would be nice to better explain why the authors think they do not have affected the associations observed (also refer to some papers that have seen an association between noise and dementia such as Yu et al Epidemiology. 2020 Nov 1;31(6):771-778). I suggest to draw a digital acyclic graph (DAG) including noise, green spaces, and other features of the urban environment to better understand their potential contribution to the studied associations.**
  - We agree with the reviewer that green space and noise are important features of the urban enrollment that need to be considered in air pollution studies, which is why we had discussed these in our limitations section. We are aware of studies suggesting that increased noise pollution may be positively associated with dementia risk, while greenspace may lower dementia risk, although the evidence is scant and inconsistent. Furthermore, noise and air pollution may be positively correlated, while green space and air pollution may be negatively correlated. Therefore, had we looked at the exposure effects of air pollution on cognitive decline, this unmeasured confounding may have potentially biased our estimated associations.
  - However, our study is focusing on the *change* in air quality over time. In this regard, it is important to evaluate the *change* in noise exposure and the *change* in greenspace concurrent with air quality improvement. The reason we believe our findings are likely not confounded by these data is because noise levels have been increasing in urban areas over this same time period (95% of participants lived in urban area at start of WHI CT). Additionally, over the years greenspace has been declining due to urbanization and development. In order to confound our results, noise levels would need to decrease/get worse and greenspace would need to increase/improve over the time span that we defined air quality improvement.
- **I am missing a table with a description of the outcomes and the number of interviews performed for each woman (I think this is not mentioned in the manuscript; also report the mean and SD in the text of the number of interviews). It would be also informative to show the distribution of each test in each interview.**
  - We added a supplementary table (S3 Table) summarizing the sample size, mean and standard deviation of cognitive tests (TICSm and CVLT) at each visit.
  - We summarized the number of interviews performed for each women in the Results section (2^nd^ paragraph) as below: “*During a median 6.2 (**interquartile range [IQR] = 5) years of follow-up, women had a median 7 (IQR = 5) interviews for TICSm tests and 6 (IQR = 3) interviews for CVLT tests. The means of the cognitive test scores did not differ much over the visits (S3 Table), while significant cognitive declines (TICSm slope = -0.42/year; 95% CI: -0.44, -0.40; CVLT slope = -0.59/year; 95% CI: -0.64, -0.54) were observed (Fig 2).*”
- Have the authors checked whether the number of interviews affected the association? (major number of interviews ◊ stronger associations?).
  - We conducted the suggested analyses testing whether the association between AQ improvement and slower cognitive decline may differ by the total number of interviews. The results showed that none of these interactions were statistically significant (interaction p-value >0.05 for both TICSm and CVLT and for AQ improvement on both PM_2.5_ and NO_2_). Therefore, the results suggest that the number of interviews did not affect the associations found.
- A table including the scores at baseline and the scores in the last interview would be also an asset and comparing this (or the distribution of the outcomes) with women's characteristics (age, BMI, education etc).
  - We added a supplementary table (S4 Table) for the distribution of scores at baseline and scores at the last interview by population characteristics.
  - The corresponding results were also added in the Results section (2^nd^ paragraph) accordingly as follows: “*Non-Hispanic Black women or women older than 80 had lower mean cognitive scores at baseline for both TICSm and CVLT (S4 Table). Women residing in the Midwest and who had higher education or income, were currently employed, currently drinking alcohol, did not have hypertension, and did not carry the ApoE e4 genotype had higher mean cognitive scores on both TICSm and CVLT at baseline, compared to their counterparts (S4 Table). The distributions of cognitive scores at the last visit were similar across these population characteristics, except that some differences were no longer significant (S4 Table).*”
- **I would include the results of the imputed dataset as main results and move the complete case ones to the supplementary material.**
  - We are happy that the reviewer was pleased with our sensitivity analysis applying multiple imputation to address missing data on AQ measures or covariates.
  - However, the use of multiple imputation is controversial and is not without pitfalls [8]. Therefore, we prefer to keep the results of the imputed dataset as a sensitivity analysis and use the complete case analysis as the main results.

- **It is not clear to me how the authors have selected the confounders since some of them are not associated with air quality improvement as shown in Table 1 (if the based on the association with exposure and outcome) and some can act as potential mediators (e.g. physical activity can mediate the association between urban design - air pollution - physical activity - dementia; cardiovascular health since some of the indicators such as obesity have been associated with air pollution exposure; or mental health). The DAG can help in visualizing which covariates act as confounders and which one can act as potential mediators or effect modifiers.**
- These aforementioned predictors could be considered confounders because they may be important deciding factors on where elderly individuals may choose to live at the WHI inception or move during the follow-up (e.g., moving for better access to healthcare; moving to live near family) and thus would potentially influence air pollution levels.
- Although *changes* in the lifestyles and cardiovascular risks may be in the causal pathways linking air quality improvement with health benefits in general, in our specific study these above-mentioned variables are likely not in the causal pathway because of temporal ordering. For the majority of women in our cohort, their 3-year average remote exposure time window was 1997-1999 and their 3-year average recent exposure time window was 2007-2009. The predictors mentioned above were collected at WHI-CT inception, which took place between 1993 and 1998. Therefore, the predictors were mostly collected prior to the remote exposure period and thus are likely not in the causal pathway. Below is a DAG representing our study variables:

- However, in light of the Reviewer’s comment we conducted an additional analysis where we removed these covariates from the model (physical activity; cardiovascular outcomes [CVD; hypercholesterolemia; hypertension; diabetes mellitus; BMI]) and have included the results below for the reviewer’s interest. The results without adjustment of these covariates were identical to those from our fully adjusted models for TICSm outcome (Model III, Table 3) and slightly varied for CVLT outcome (β_PM2.5 improvement_ = 0.071, 95% CI=0.02, 0.12; β_NO2 improvement_ = 0.060, 95% CI=0.006, 0.12).
- We would also like to let the Reviewer know that we have restructured our models in Table 3 (originally Table 2) as follows:
  - We kept our Model I the same, which included the essential set of covariates that needed to be adjusted for in our models to examine the average decline rates in the TICSm and CVLT trajectories during the follow-up and to account for spatiotemporal confounding and selective attrition over the study period. We refer to this as our base model.
  - We have added a new model to Table 3, which is referred to as the new Model II. This model included the covariates in Model I plus any variables that researchers in the field would agree to be predictors of both air pollution exposure and brain aging (e.g., geographic region; race/ethnicity; socioeconomic factors [education, income, employment status] and neighborhood socioeconomic characteristics; lifestyle factors [smoking, drinking; physical activities]).
  - Our original Model II has now been renamed to Model III. This Model III included the covariates in Model II plus any variables that some researchers may argue could theoretically be mediators of the pathway between air pollution exposure and brain aging, as you highlighted above (e.g. prior hormone use; hormone therapy assignment; depression; cardiovascular risk factors [hypertension, diabetes and hypercholesterolemia], body mass index; cardiovascular disease histories). We refer to this as our fully adjusted model.
  - Our results indicate that the estimated associations did not vary much across models.
- **In figure 3B it seems that BMI modifies the association between air quality but this is not mentioned.**
- The p-values presented in Figure 3 were estimated from each model individually without controlling for multiple comparison. After controlling for multiple comparison using Benjamini–Hochberg approach, False Discovery Rate corrected p-values > 0.05 for all interaction tests. We included this information in the figure footnote.
- **Clinical sites are firstly mentioned in the statistical analysis. A good description of them should be included in the study population and also in Table 1 as another population characteristic.**
- We have created a new figure (S1 Figure) which is a U.S. map of where the clinic sites are located. In the Methods section (1^st^ paragraph), we also added the following: *“WHIMS participants (N=7479) were community-dwelling postmenopausal women without dementia (aged ≥65 years) who resided in the 48 contiguous U.S. states and were recruited from more than 40 study sites located in 24 states and Washington, D.C.*
- By suggesting the information of clinic sites included in Table 1, the reviewer seemed to be interested in the possible difference in air quality measures by clinic site. We agree it is scientifically interesting to look at the local-scale difference in AQ improvement, beyond what was already reported by the geographic region where WHIMS participants resided at the baseline.
- Unfortunately, in accordance with WHI guidelines, we are unable to disclose any information on geographical identifiers below the state level.
- Additionally, looking at the distribution of air quality measures by clinic site would not be appropriate for a couple of reasons. First, although participants resided across 48 states, they were recruited from more than 40 study sites across 24 U.S. states and Washington, DC. Second, some participants could have moved before WHIMS-ECHO enrollment and looking at their clinic centers could give a false impression that some localities had better AQ improvement than others, while the exposure contrast in fact might have been driven by relocations and temporal changes not specific to the regional/local centers. Third, because of the large geographic coverage of WHI cohort, some participants went across state borders to participate in the WHI follow-up. These women may have attended a clinic within their state at WHI-CT, but then chose to attend a clinic in another state during follow-up. Therefore, using the in-state clinic center at WHI enrollment may not be an accurate representation for those women who chose to attend a clinic in another region between WHI-CT and WHIMS-ECHO.
- **I would include the association between remote and recent NO2 and PM2.5 exposure with each of the outcomes as main analysis and then report the air quality improvement in the same table similar to Table S5 (very difficult to understand what each Models mean).**
- In our study, the primary exposure of interest is the air quality improvement which was defined as the reduction of air pollution from remote (10 years earlier) to recent (at WHIMS-ECHO enrollment) time points before the outcome assessment started. Numerous studies [9], including our own [10-12], have reported on the exposure effects of air pollution on cognitive decline, but none have explored whether a reduction in air pollution may also be associated with a slower decline in cognitive function. The purpose of this study was to address this gap in the literature.
  - Our reason for including recent and remote PM_2.5_ and NO_2_ in our models was to explore whether our findings could be explained by regression to the mean in air quality measures. Therefore, we prefer not to report the exposure effects of PM_2.5_ and NO_2_ as the main analysis and prefer to keep these results separate.
  - The original S5 Table (now renamed as S7 Table) presented the associations between AQ improvement, recent exposure, or remote exposure with cognitive trajectory slopes, with each individual exposure included in Models I, II and III, respectively. The associations that were estimated from Model IV had both AQ improvement and recent exposure included in the same model, while associations estimated from Model V had both AQ improvement and remote exposure included in the same model. The purpose of Models IV and V was to explore the potential impact of regression to the mean in air quality measures on the association between AQ improvement and cognitive trajectory slopes.
  - We edited the footnote of S7 Table to clarify: “*^d^ Model I included air quality improvement measure only. Model II included recent air pollution exposure only. Model III included remote air pollution exposure only.*

*^e^ Model IV i~~I~~ncluded both air quality improvement and recent exposure in the same model.*

*^f^ Model V i~~I~~ncluded both air quality improvement and remote exposure in the same model.*”

- **Please, discuss how the estimation of air pollution can have varied throughout the 10 years period and how this may have affected the results (and perform some analyses if needed).**
  - Although the air pollution estimation models we used performed well (cross-validated R^2^ consistently over 0.85) and both correlation and RMSE-based performance statistics are reasonably consistent across these time periods, we acknowledge that the errors in predicting air pollution estimates may have varied throughout the 10-year period and the potential measurement errors could have biased our results.
  - We added this as one limitation in the Discussion (5^th^ paragraph) as follows: “*Third, the errors in predicting the air pollution estimates may have varied throughout the 10-year period and may have contributed uncertainty to our analyses.*”

**Minor comments**

- **Introduction and discussion - it would be nice to discuss some equivalent analysis between air pollution and cognition development in children such as the study conducted in Barcelona schools (PLoS Med. 2015 Mar 3;12(3):e1001792).**
  - We have added the following in the Discussion (3^rd^ paragraph): “*Lastly, our findings may provide the impetus for future research to consider how AQ improvement could potentially benefit brain development, as studies have found that higher levels of air pollution may hinder cognitive development in children [61].”*
- **How deaths are treated in the analysis?**
  - We did not use death information specifically in our analyses. We adjusted for time-varying propensity scores in our analyses to control for attrition due to loss-of-follow-up. It is possible that some women were lost to follow-up due to death.
- **Use the same wording in the text and in Figure 1 regarding the outcomes assessed.**
  - To be consistent with Figure 1 regarding the outcomes assessed, we revised the Methods (Study design and population subsection) as follows: “*We excluded 346 women with prevalent dementia at WHIMS-ECHO enrollment or without follow-up visits, resulting in a sample of 2534 women with at least 2 modified Telephone Interview for Cognitive Status (TICSm) measures of general cognitive status (Fig 1A). For the analyses on episodic memory decline, we further excluded 587 women without repeated measures of episodic memory assessed by ~~cognitive measures~~California Verbal Learning Test (CVLT)… This resulted in a final analytic sample of 2232 women for the analyses on general cognitive status assessed by TICSm, a subset of which (n=1721) was used for the analyses on episodic memory assessed by CVLT (Fig 1A).*”
- **I would not say 'Older women' in sentences 216 and 230 because it seems the authors are comparing women within the study based on their age. I would just say 'Women'.**
  - We have changed these to just say “women”.
- **Include the confounding variables in the models in Figure 2 footnote.**
  - We have included the confounding variables in the models in Figure 2 footnote as follows: “*The estimated TICSm scores or CVLT scores were calculated using parameter estimates derived from Model III of Table 3, which were adjusted for spatial random effect, WHIMS-ECHO enrollment year, age, follow-up year, age interaction with follow-up year, geographic region, race/ethnicity, education, income, employment status, neighborhood socioeconomic characteristics, smoking, drinking, physical activities, prior hormone use, hormone therapy assignment, hypertension, diabetes, hypercholesterolemia, depression, body mass index, cardiovascular disease histories, and time-varying propensity scores.*”
- **In table 2, I would change Model I and Model II for Minimally adjusted model and Fully adjusted model. If authors select confounders based on the DAG, then the minimally adjusted model is no longer needed.**
  - As previously mentioned, our Table 3 (originally Table 2) now includes 3 models, with Model I representing our base model and Model III representing our fully adjusted model.
  - We prefer to refer to these as Model I, Model II, and Model III. The reason for this is because researchers in different fields may use different terminology for describing these models which may make it a bit more complicated.
  - In the revision, however, we did specify when we were referring to our fully adjusted model as follows: *“Based on the fully adjusted models (Table 3, Model III), ~~For~~ each IQR increment of improved AQ in PM_2.5_ and NO_2_ (IQR_PM2.5_ = 1.79 µg/m^3^ for both analytic samples; IQR_NO2_ = 3.92 ppb for TICSm and 3.97 ppb for CVLT analyses) ~~in the fully adjusted models (Table 2, Model II),~~ was associated with diminished ~~the observed~~ cognitive declines over time ~~were diminished~~ by 0.026-0.034/year in TICSm…”* (Results, 3^rd^ paragraph)

**Reviewer #3: This article reports a detailed evaluation of whether improvement in air quality is associated with cognitive change in older women using the Women's Health Initiative study dataset. The authors have linked this to geocoded measures of environment and air quality over time. Air pollution is a risk factor for cognitive decline and dementia, so this is an important study because it evaluates whether risk reversal is associated with any cognitive benefit. The investigation is rigorous and well considered. Authors are careful not to claim causality as the study is observational and it is possible that there are unmeasured confounders. There are some areas of the methodology that were unclear such as:**

1. **It is unclear how exposure to air pollution over time was estimated at the level of the individual participant, particularly when they moved residence during the study. Can the authors please explain in sufficient detail for full replication of methods, exactly how an individual's 'air pollution reduction' score was derived?**
   - Participants’ residential addresses were prospectively collected at each WHI assessment since its inception in 1993, updated at least biannually. The exact date of address change was used in analyses when available, otherwise the date when the change in residence was ascertained was used.
   - At each residential address, the annual mean air pollution estimates were calculated. These annual estimates were then used to aggregate over the 3-year time window prior to the WHIMS-ECHO enrollment (referred to as recent exposure) or at 10 years earlier (referred to as remote exposure). The residential mobility was taken into account by using the length of stay in days at each residential location during each 3-year exposure time window as the weight in the calculation.
   - The difference between the remote and recent exposures was then used to indicate long-term air pollution reduction over the 10 years prior to WHIMS-ECHO enrollment for each woman.
   - We revised the Methods (Estimation of air pollution exposure subsection) to make it clearer: “*Participants’ residential addresses were prospectively collected at each WHI assessment since its inception in 1993, updated at least biannually, and then geocoded using standardized procedures [33]. The exact date of address change was used in analyses when available, otherwise the date when the change in residence was ascertained was used. We used validated regionalized national universal kriging models with partial least squares regression of geographic covariates and US Environmental Protection Agency (EPA) monitoring data to estimate annual mean concentrations of PM_2.5_ (in μg/m^3^) and NO_2_ (in ppb; a proxy of traffic-related air pollutants) at each of these residential addresses~~, accounting for residential mobility over time~~… These annual estimates were then aggregated to the 3-year average at the WHIMS-ECHO enrollment (referred to as recent exposure) and the corresponding 3-year average 10 years earlier (referred to as remote exposure), accounting for residential mobility within each 3-year time window. The individual-level measure of long-term AQ improvement over the 10-year period was defined as the reduction from remote to recent exposures (Fig 1B).*”
2. **What is the validity and reliability of the environmental measures and air pollution measures that were used in this study?**
   - The environmental measure used in this study is neighborhood SES. We added the following sentences in the S2 Appendix to describe its validity and reliability: “*Data from the U.S. Census of Population and Housing 2000 Summary File 3 [1] or their 5-year analogs from the American Community Survey 2005-2009 to 2013-2017 [2]) were temporally matched to the geocoded participant addresses [3,4]). Each measure had been aggregated at the U.S. Census tract level (i.e., the lowest geographic level historically associated with accurate and reliable assignment of Federal Information Processing System codes) [4].*”
   - The annual estimates of air pollution exposures were generated using validated models that have high cross-validation R^2^ (0.88 for PM_2.5_ and 0.85 for NO_2_). In aggregating 3-year average air pollution measures using these annual estimates, we accounted for the residential mobility by using the length of stay at each location within the 3-year time window as the weight in calculation. More detailed information has been included in the Methods, Estimation of air pollution exposure section.
3. **Did the authors take into consideration where participants spent their time during each day or did they only examine residential location of participants? for example, an individual may live in one area but work in another and effectively spend more time away from their residential location.**
   - In our analyses, we only estimated air pollution levels at residential locations of participants. This approach did not consider an individual’s time-activity pattern which however could be varying over time and not reliably assessed during the selected exposure time windows. Given the majority (~84%) women in our study sample were retired or not working, we think the situations that they spent more time (e.g., at work in a different location) away from their residential location were not common.
   - In the discussion section, we had already acknowledged this limitation as follows: “*Second, we estimated the ambient levels of air pollution at residential locations* *and detailed information on time-activity patterns were not available ~~did not incorporate personal exposure information~~. However, the use of individual-level estimates of reduced exposures to ambient air pollutants~~ion exposures~~ is appropriate for studying the public health benefits [23,25-27] since these pollutants are regulated by the EPA.*”
4. **When participants moved, how was time treated in the statistical modelling. i.e what was the unit of time - was it days, weeks, months or a year?**
   - As we described in detailed in the response to comment #1 above, the exact date of residential address change was used in analyses when available, otherwise the date when the change was ascertained was used. The length of stay in days at each residential location during each 3-year exposure time window was used as the weight in aggregating 3-year average air pollution exposures at both recent and remote time points.
5. **The geographical regions would not be well understood outside of the USA. it is suggested that these are better described in the method or supplement. Similarly the education levels are not described in widely understood terms. In the US 'College' refers to what other countries call 'University' or 'tertiary' level education. To ensure wider relevance it would be useful to define this variable more clearly.**

- We have added a new figure in the supplement (S1 Figure) that clearly defines the geographical regions.
- Regarding education levels, we defined these according to the WHI data dictionary.

6. **More information is needed on the APOE genotyping and how this variable was classified and treated in statistical analyses as there are different practices in the literature eg. some studies exclude certain combinations of e2, e3, and e4 alleles or combine e4 homozygous and e4 heterozygous. APOE e4 status should also be reported in the descriptive statistics.**

- We have added the distribution of ApoE into Table 1 and Table 2.
- We also added more information in S6 Appendix (APOE genotype data) as follows: “*Women with genotypes e2/2, e2/3 and e3/3 were combined as one group representing those without e4 allele, while women with genotypes e2/4, e3/4 and e4/4 were grouped together representing women with at least one copy of e4 allele in analysis.*”

7. **Can the authors provide any information on how the missing data on the CVLT would have biased results?**

- CVLT was not implemented in the phone interview at the beginning of WHIMS-ECHO and was added 1-2 years later. Therefore, the analytic sample for CVLT outcome excluded women who were lost to follow-up before the CVLT was added to the annual assessment.
- Comparing to those excluded due to not having repeated CVLT measures (Figure 1A), women with repeated CVLT measures were more likely to be younger, have higher education and income, currently drink alcohol, without hypertension, and without ApoE e4 genotype. These results are summarized in a new S1 Table.
- We adjusted for these covariates to control for potential confounding in our analyses on CVLT outcome. In addition, we adjusted for time-varying propensity scores in all models to account for selective attrition over the WHIMS-ECHO follow-up. We could not completely rule out the possible bias due to exclusion of these women in our study though.
- We added the following sentences in the Results: “*Compared to those excluded due to not having repeated CVLT measures (Figure 1A), women with repeated CVLT measures were more likely to be younger, have higher education and income, and currently drink alcohol, but less likely to report having hypertension and carry the ApoE e4 genotype (S1 Table).*” (Results, 1^st^ paragraph)
- We added this as one limitation in the Discussion (5^th^ paragraph) as follows: “*Sixth, because our study sample came from a non-random selective process (Figure 1A), we could not completely rule out the possibility of selection biases.*”

8. **Were these the only two cognitive measures collected by the WHIMS study? Other papers mention the 3MSE which is an extended Mini-Mental State Examination.**

- The reviewer is correct that other WHIMS papers have mentioned the Modified Mini Mental State (3MS). Between 1996 and 2008, women completed the 3MS examination each year. However, beginning in 2008 when WHIMS-ECHO enrollment began, annual cognitive screenings were conducted by telephone using the Telephone Interview for Cognitive Status-modified. To ensure temporality between AQ improvement and cognitive decline, we did not include cognitive assessments prior to WHIMS-ECHO enrollment which is why we did not use 3MS assessments in this study.
- We specifically focused on general cognitive status and episodic memory for a couple of reasons. The majority of previous studies on air pollution and cognitive decline focused on general cognition function, which is why we wanted to include general cognitive status. We also wanted to explore the association between AQ improvement and episodic memory because this cognitive domain is one of the most sensitive cognitive indicators for early detection of AD. Lastly, declines in both measures had been found to be associated with PM_2.5_ in WHIMS participants in our previous studies [10-12].

References

1.	Rosenman R, Tennekoon V, Hill LG. Measuring bias in self-reported data. Int J Behav Healthc Res. 2011;2(4):320-32. doi: 10.1504/IJBHR.2011.043414. PubMed PMID: 25383095; PubMed Central PMCID: PMCPMC4224297.

2.	Rapp SR, Legault C, Espeland MA, Resnick SM, Hogan PE, Coker LH, et al. Validation of a cognitive assessment battery administered over the telephone. J Am Geriatr Soc. 2012;60(9):1616-23. doi: 10.1111/j.1532-5415.2012.04111.x. PubMed PMID: 22985137; PubMed Central PMCID: PMCPMC3448122.

3.	Pope CA, Ezzati M, Dockery DW. Fine-particulate air pollution and life expectancy in the United States. N Engl J Med. 2009;360(4):376-86. doi: 10.1056/NEJMsa0805646. PubMed PMID: 19164188; PubMed Central PMCID: PMCPMC3382057.

4.	Laden F, Schwartz J, Speizer FE, Dockery DW. Reduction in fine particulate air pollution and mortality: Extended follow-up of the Harvard Six Cities study. Am J Respir Crit Care Med. 2006;173(6):667-72. Epub 2006/01/19. doi: 10.1164/rccm.200503-443OC. PubMed PMID: 16424447; PubMed Central PMCID: PMCPMC2662950.

5.	Avery CL, Mills KT, Williams R, McGraw KA, Poole C, Smith RL, et al. Estimating error in using residential outdoor PM2.5 concentrations as proxies for personal exposures: a meta-analysis. Environ Health Perspect. 2010;118(5):673-8. Epub 2010/01/14. doi: 10.1289/ehp.0901158. PubMed PMID: 20075021; PubMed Central PMCID: PMCPMC2866684.

6.	Sheppard L, Burnett RT, Szpiro AA, Kim SY, Jerrett M, Pope CA, et al. Confounding and exposure measurement error in air pollution epidemiology. Air Qual Atmos Health. 2012;5(2):203-16. Epub 2011/03/23. doi: 10.1007/s11869-011-0140-9. PubMed PMID: 22662023; PubMed Central PMCID: PMCPMC3353104.

7.	Chen JC, Schwartz J. Neurobehavioral effects of ambient air pollution on cognitive performance in US adults. Neurotoxicology. 2009;30(2):231-9. Epub 2008/12/30. doi: 10.1016/j.neuro.2008.12.011. PubMed PMID: 19150462.

8.	Sterne JAC, White IR, Carlin JB, Spratt M, Royston P, Kenward MG, et al. Multiple imputation for missing data in epidemiological and clinical research: potential and pitfalls. BMJ. 2009;338:b2393. doi: 10.1136/bmj.b2393.

9.	Peters R, Ee N, Peters J, Booth A, Mudway I, Anstey KJ. Air Pollution and Dementia: A Systematic Review. J Alzheimers Dis. 2019;70(s1):S145-S63. doi: 10.3233/JAD-180631. PubMed PMID: 30775976; PubMed Central PMCID: PMCPMC6700631.

10.	Younan D, Petkus AJ, Widaman KF, Wang X, Casanova R, Espeland MA, et al. Particulate matter and episodic memory decline mediated by early neuroanatomic biomarkers of Alzheimer's disease. Brain. 2020;143(1):289-302. doi: 10.1093/brain/awz348. PubMed PMID: 31746986; PubMed Central PMCID: PMCPMC6938036.

11.	Petkus AJ, Younan D, Widaman K, Gatz M, Manson JE, Wang X, et al. Exposure to fine particulate matter and temporal dynamics of episodic memory and depressive symptoms in older women. Environ Int. 2020;135:105196. Epub 2019/12/24. doi: 10.1016/j.envint.2019.105196. PubMed PMID: 31881430.

12.	Cacciottolo M, Wang X, Driscoll I, Woodward N, Saffari A, Reyes J, et al. Particulate air pollutants, APOE alleles and their contributions to cognitive impairment in older women and to amyloidogenesis in experimental models. Transl Psychiatry. 2017;7(1):e1022. Epub 2017/01/31. doi: 10.1038/tp.2016.280. PubMed PMID: 28140404; PubMed Central PMCID: PMCPMC5299391.

13.	Whitsel EA, Quibrera PM, Smith RL, Catellier DJ, Liao D, Henley AC, et al. Accuracy of commercial geocoding: assessment and implications. Epidemiol Perspect Innov. 2006;3:8. Epub 2006/07/20. doi: 10.1186/1742-5573-3-8. PubMed PMID: 16857050; PubMed Central PMCID: PMCPMC1557664.

14.	Berhane K, Chang CC, McConnell R, Gauderman WJ, Avol E, Rapapport E, et al. Association of Changes in Air Quality With Bronchitic Symptoms in Children in California, 1993-2012. JAMA. 2016;315(14):1491-501. doi: 10.1001/jama.2016.3444. PubMed PMID: 27115265; PubMed Central PMCID: PMCPMC5679287.

15.	Garcia E, Berhane KT, Islam T, McConnell R, Urman R, Chen Z, et al. Association of Changes in Air Quality With Incident Asthma in Children in California, 1993-2014. JAMA. 2019;321(19):1906-15. doi: 10.1001/jama.2019.5357. PubMed PMID: 31112259; PubMed Central PMCID: PMCPMC6537847.

16.	Gauderman WJ, Urman R, Avol E, Berhane K, McConnell R, Rappaport E, et al. Association of improved air quality with lung development in children. N Engl J Med. 2015;372(10):905-13. doi: 10.1056/NEJMoa1414123. PubMed PMID: 25738666; PubMed Central PMCID: PMCPMC4430551.
