# Supplementary material for: Air quality improvement and cognitive decline in community-dwelling older women in the United States: A longitudinal cohort study
Source: PLoS Med. 2022 Feb 3;19(2):e1003893. doi: 10.1371/journal.pmed.1003893 (PMC8812844; doi:10.1371/journal.pmed.1003893)
Supplement: Attachment — Submitted filename: Response to Reviewers.docx [file pmed.1003893.s024.docx]

**Requests from Editors:

Your title should be non-declarative. Please update to ‘Air quality and cognitive decline in community-dwelling older women in the United States: A longitudinal cohort study’.**

- We have followed the Editors’ suggestion to change the title.
- However, our study focused on analyzing the impact of air quality *improvement*, so we believe it is important to include “improvement” in the title. Therefore, we have changed the title to: ‘Air quality improvement and cognitive decline in community-dwelling older women in the United States: A longitudinal cohort study’.

**The Data Availability Statement (DAS) requires revision. If the data are owned by a third party but freely available upon request, please note this and state the owner of the data set and contact information for data requests (web or email address). The URL you have provided in your statement appears to be a broken link.**

**“*Data statement: Data, codebook, and analytic code used in this report may be accessed in a collaborative mode as described on the Women’s Health Initiative website (https://urldefense.com/v3/__http://www.whi.org__;!!LIr3w8kk_Xxm!76lJ1eegY7-QWP88j12lZ9GmitZamnptj8mzyvGVq9XEfIBi-3CzQc8cO5RNwf4$ ).*”**

- We have revised this statement as follows: “*Data, codebook, and analytic code used in this report are held by the NIH-funded Coordinating Center of the Women’s Health Initiative at the Fred Hutchinson Cancer Research Center and may be accessed ~~in a collaborative mode~~ as described on the Women’s Health Initiative website: ~~(https://urldefense.com/v3/__http://www.whi.org__;!!LIr3w8kk_Xxm!76lJ1eegY7-QWP88j12lZ9GmitZamnptj8mzyvGVq9XEfIBi-3CzQc8cO5RNwf4$ )~~ https://www.whi.org/page/working-with-whi-data.*”

**Line 53: Please update to ‘in older women aged 74-92 years’.**

- We have changed this sentence as suggested.

**Please include basic information about the nationwide remit of the WHIMS-ECHO cohort in your abstract methods and findings (i.e. ‘Participants resided in the 48 contiguous U.S. states and were recruited from more than 40 study sites located in 24 states and Washington, D.C.’).**

- We edited the sentence as below to incorporate the information: “*~~We studied a geographically-diverse cohort of predominantly non-Hispanic White women from~~We studied a cohort of 2232 women residing in the 48 contiguous U.S. states that were recruited from more than 40 study sites located in 24 states and Washington, D.C. from the Women’s Health Initiative Memory Study-Epidemiology of Cognitive Health Outcomes (WHIMS-ECHO). ~~who~~ They were predominantly non-Hispanic White women and were dementia free at baseline in 2008-12 ~~(N=2232, aged 74-92)~~.*”

**Your Author Summary should not recycle text from the abstract as far as is possible, please revisit and ensure you have not copied text directly across (e.g. bullet point 1).**

- We have changed the Author Summary as follows:
  - “Although studies have shown that *~~Ll~~ate-life exposure to outdoor air pollution is a modifiable risk factor for dementia, ~~but~~ the epidemiological ~~studies have shown inconsistent~~ evidence for cognitive decline has been inconsistent.*”
  - “*Using a U.S. cohort of 2232 older women followed up to 20 years, we explored whether women living in locations with greater air quality improvement ~~were less likely to~~ had slower decline in their cognitive function.*”

**Please consider relocating some of the text describing your study population from the methods to the results section. The methods section ought to describe the WHIMS study and define inclusion/exclusion criteria, while the final analytic sample and its demographic characteristics would be better placed in the results.**

- We have moved the detailed text regarding the final analytic sample to the results section and also moved Figure 1 so that it appears after this text.
- In the Methods, we added a general description of our inclusion/exclusion criteria as follows: *“The analyses were restricted to women without prevalent dementia at WHIMS-ECHO enrollment and with follow-up visits and complete data on AQ measures and relevant covariates.”*

**Your rebuttal letter notes that you updated some of your analyses based on reviewer comments. Changes in the analysis, including those made in response to peer review comments, should be identified as such in the Methods section of the paper, with rationale (this can be included in the Study design and population subsection).**

- We have added the following text in the Methods (*Study design and population* subsection) to highlight the changes made in our analysis as a result of the peer review process: *“During the peer review process, we added a partially adjusted model in our main analyses and ad hoc analyses to explore the potential impact of non-linear cognitive trajectory and non-linear AQ improvement effects on cognitive trajectory slope.”*

**Line 360: Ensure footnote ‘f’ begins on a new line.**

- We have made this change.

**Line 467: Please update ‘studies’ to ‘at least one study’ as you only cite one study.**

- We retained the sentence as is, and instead cited a systematic review that summarizes studies that evaluated the relationship between air pollution and cognition in children.

**Please remove your ‘Financial disclosure’, ‘Competing interests’, and ‘Data statement’ from the end of the main text; all of this information will be captured as metadata based on your responses to the submission form questions (so please ensure all relevant information is included in your answers).**

- We have removed these sections from the main text.

**Similarly, the ‘Ethical approval’ statement (line 545) can be removed as this is already included in your Methods section.**

- We have removed this section from the main text.

**Please check the formatting of reference 50.**

- We have updated this reference.

**You have included a map in your updated supplementary materials. Please confirm that the appropriate usage rights apply to the use of this map. Please see our guidelines for map images:** [**https://urldefense.com/v3/__https://journals.plos.org/plosmedicine/s/figures*loc-maps__;Iw!!LIr3w8kk_Xxm!532fLHi2yzFh3tNREiBiufrD1UNzjDN6q7bHRNfkxRtTUiKO1auNtJVnMxsvfbs$**](https://urldefense.com/v3/__https:/journals.plos.org/plosmedicine/s/figures*loc-maps__;Iw!!LIr3w8kk_Xxm!532fLHi2yzFh3tNREiBiufrD1UNzjDN6q7bHRNfkxRtTUiKO1auNtJVnMxsvfbs$)

- We have double checked the usage rights of this map.
- According to PLOS Medicine’s guidelines for map images, this map is compatible with CC BY licensing since it is a USGS map that is in the public domain.
- Additionally, the map was created via ArcMaps and the base layer was obtained from the ArcGIS website (<https://www.arcgis.com/home/item.html?id=99fd67933e754a1181cc755146be21ca>). According to the ArcGIS display copyrights (https://doc.arcgis.com/en/arcgis-online/reference/display-copyrights.htm), it says to copy and paste the attribution at the bottom of the map, which we have done.

**Comments from Reviewers:

Reviewer #1: The authors have satisfactorily considered and responded to each comment in turn, providing clarifications and amending the manuscript where required.


Reviewer #2: The authors properly replied to all my concerns. I do not have any other additional comment.


Any attachments provided with reviews can be seen via the following link:

__________________________________________________
In compliance with data protection regulations, you may request that we remove your personal registration details at any time.  (Use the following URL:** [**https://urldefense.com/v3/__https://www.editorialmanager.com/pmedicine/login.asp?a=r__;!!LIr3w8kk_Xxm!532fLHi2yzFh3tNREiBiufrD1UNzjDN6q7bHRNfkxRtTUiKO1auNtJVnN-slOr0$**](https://urldefense.com/v3/__https:/www.editorialmanager.com/pmedicine/login.asp?a=r__;!!LIr3w8kk_Xxm!532fLHi2yzFh3tNREiBiufrD1UNzjDN6q7bHRNfkxRtTUiKO1auNtJVnN-slOr0$) **). Please contact the publication office if you have any questions.**
